# Efficient perovskite/Cu(In,Ga)Se_2_ tandem solar cells with a composite intermediate recombination layer

**DOI:** 10.1038/s41467-025-67350-y

**Published:** 2025-12-10

**Authors:** Wang Li, Junjun Zhang, Li Zeng, Wanhai Wang, Zhou Fang, Xinxing Liu, Zengyang Ma, Yitian Zhang, Hui Yan, Chen Shen, Zhuo Xue, Jingyi Zhu, Qiren Luo, Chang Liu, Ruixuan Jiang, Tongle Bu, Weihua Tang, Jianmin Li, Sheng Wang, Junbo Gong, Xudong Xiao

**Affiliations:** 1 School of Physics and Technology, Key Lab of Artificial Micro- and Nano-structures of Ministry of Education, Wuhan University, Wuhan, 430072 Hubei China; 2 Institute of Flexible Electronics (IFE Future Technologies), College of Materials, Innovation Laboratory for Sciences and Technologies of Energy Materials of Fujian Province (IKKEM), Xiamen University, Xiamen, 361005 China; 3 State Key Laboratory of Advanced Technology for Materials Synthesis and Processing, Wuhan University of Technology, Wuhan, 430070 PR China

**Keywords:** Solar cells, Solar cells

## Abstract

Monolithic perovskite/Cu(In,Ga)Se_2_ (CIGS) tandem solar cells offer unique advantages among tandem configurations, including optimal bandgap pairing, all-thin-film architecture, superior radiation hardness, and exceptional stability. Despite their potential, challenges remain in optimizing efficiency and stability, particularly in the intermediate recombination layer (IRL) that connects subcells. This study addresses these challenges by developing a high-performance IRL using a composite structure comprising Al:ZnO/Au/NiO_x_/[4-(7H-dibenzo[c,g]carbazol-7-yl)butyl] phosphonic acid (4PADCB). The hybrid NiO_x_/4PADCB hole transport layer enhances interface trap passivation, optimizes band alignment, and improves minority carrier extraction, while the ultrathin Au layer significantly boosts the majority carrier recombination rate. With well-engineered subcells, our champion perovskite/CIGS tandem device achieved a record PCE of 28.04% at 0.51 cm^2^ area and 30.71% at 0.15 cm^2^ area (30.1% cross-verified by an external organization), with an exceptional fill factor of 80.9%, alongside outstanding photo- and thermal-stability. This work establishes monolithic perovskite/CIGS tandems as competitive with leading perovskite/Si (34.9%) and perovskite/perovskite (30.1%) technologies, providing a scalable, versatile framework for next-generation photovoltaics.

## Abstract (web summary)

Challenges remain in optimizing efficiency and stability of monolithic perovskite/Cu(In,Ga)Se_2_ (CIGS) tandem solar cells. Here, authors develop a composite-structured intermediate recombination layer and achieve maximum efficiency of 28.04% and 30.71% for 0.51 cm^2^ and 0.15 cm^2^ cells, respectively.

## Introduction

Perovskite-based tandem solar cells are emerging as a leading photovoltaic technology due to their potential for achieving high power conversion efficiency (PCE) at low production costs. Among the various options for narrow-bandgap bottom subcells, including silicon^1–5^, CIGS^6–11^, and low-bandgap perovskite^12–15^, the perovskite/Si tandem is the most extensively studied and has achieved a record PCE of 34.9%^16,17^. In contrast, the perovskite/CIGS tandems present unique advantages over their counterparts. With tunable CIGS band gaps as low as 1.00 eV, they can provide a bandgap pairing closest to the theoretical requirement: 1.62 eV for the top cell and 0.96 eV for the bottom cell to obtain the maximum theoretical efficiency^18^. As an all-thin-film technology, they possess a device architecture compatible with flexible and lightweight photovoltaic applications^6^. Moreover, the radiation hardness of both subcells makes them particularly well-suited for space applications^19,20^.

A number of studies have focused on enhancing the performance of two-terminal monolithic perovskite/CIGS tandems, particularly addressing the intermediate recombination layer (IRL) that connects the two subcells^6,9,11,21^. Early work employed a chemical-mechanical polished indium tin oxide (ITO) layer to mitigate the rough surface of the CIGS subcell, achieving a certified PCE of 22.4%^6^. To avoid surface damages introduced by polishing, subsequent work applied conformal thin NiO_x_ layers or self-assembled monolayers (SAMs), such as [2-(3,6-dimethoxy-9H-carbazol-9-yl)ethyl]phosphonic acid (MeO-2PACz) and [4-(3,6-dimethyl-9H-carbazol-9-yl)butyl]phosphonic acid (Me-4PACz), directly on the as-grown transparent conducting oxide surface of CIGS subcells. These approaches achieved certified PCEs of 21.6%^9^, 23.3%^10^, and 24.2%^11^ on rigid substrates. In the meantime, notable progress has also been made on flexible perovskite/CIGS tandem solar cells, reaching efficiencies of 23.64% and 23.28%^22,23^. The most recent advance used a NiO_x_/[2-(9H-carbazol-9-yl)ethyl]phosphonic acid (2PACz) double layer on a relatively smooth CIS subcell, yielding a record PCE of 24.9% (certified 23.5%)^21^ on rigid substrates, and a NiO_x_/4PADCB configuration enabled a flexible perovskite/CIGS tandem to reach 22.8%^24^, nearly concurrent with this work. Despite these achievements, the progress of monolithic perovskite/CIGS tandems remained far behind perovskite/Si tandems (34.9%) and perovskite/perovskite tandems (30.1%)^16,17^. Notably, over the course of nearly 8 years, the efficiency of monolithic perovskite/CIGS tandems improved slightly from 22.4%^6^ (2018), 24.2%^11^ (2022), to 26.3%^16^ (2025), reflecting the tremendous challenges that remain in pushing this technology forward. These challenges primarily stem from non-optimized subcell efficiencies, mismatched band gaps, and, critically, the lack of efficient IRLs (Supplementary Table 1).

To address these limitations, we developed a composite IRL consisting of Al:ZnO(AZO)/Au/NiO_x_/4PADCB for monolithic perovskite/CIGS tandems. The multi-layer HTL was designed to leverage the suitable work function and optoelectronic properties of NiO_x_^25^, which conform well to the rough surface of the CIGS bottom subcell to suppress microscale shunt paths. The addition of 4PADCB not only passivates NiO_x_ surface defects, reduces interfacial recombination, and enhances hole extraction through favorable energy level alignment, but also facilitates high-quality perovskite film formation owing to its excellent solubility, wetting behavior, and large dipole moment compared to other SAMs.^15,26,27^ An ultrathin Au layer is further introduced between the subcells to promote efficient majority carrier recombination. Notably, this composite IRL can be directly applied to CIGS subcells without requiring surface polishing. As a result, we achieved perovskite/CIGS tandems with high open-circuit voltage (*V*_OC_, 1.74 V), substantial short-circuit current density (*J*_SC_, 21.76 mA/cm^2^), and an exceptional fill factor (FF, 80.90%). The champion device, with an active area of 0.15 cm^2^ and featuring a 1.01 eV bandgap bottom subcell and a 1.67 eV bandgap top subcell, achieved a PCE of 30.71% (30.10% cross-verified by an external organization). This represents a remarkable absolute increase of nearly 4% improvement over the previous certified record of 26.3%^16^, making it another 2-terminal tandem with efficiency exceeding 30%. Our achievement places perovskite/CIGS tandems alongside leading technologies like perovskite/Si (34.9%) and perovskite/perovskite (30.1%), as shown in the NREL chart^16^. Furthermore, the composite IRL suppresses photo-induced segregation in the perovskite layer and enhances the stability of unencapsulated tandem devices. Overall, this design not only significantly improves efficiency and stability but also offers a versatile framework that can be adapted to other tandem systems, broadening its potential impact on high-efficiency photovoltaic technologies.

## Results and discussion

### Construction of an ideal hybrid HTL

**Fig. 1:**
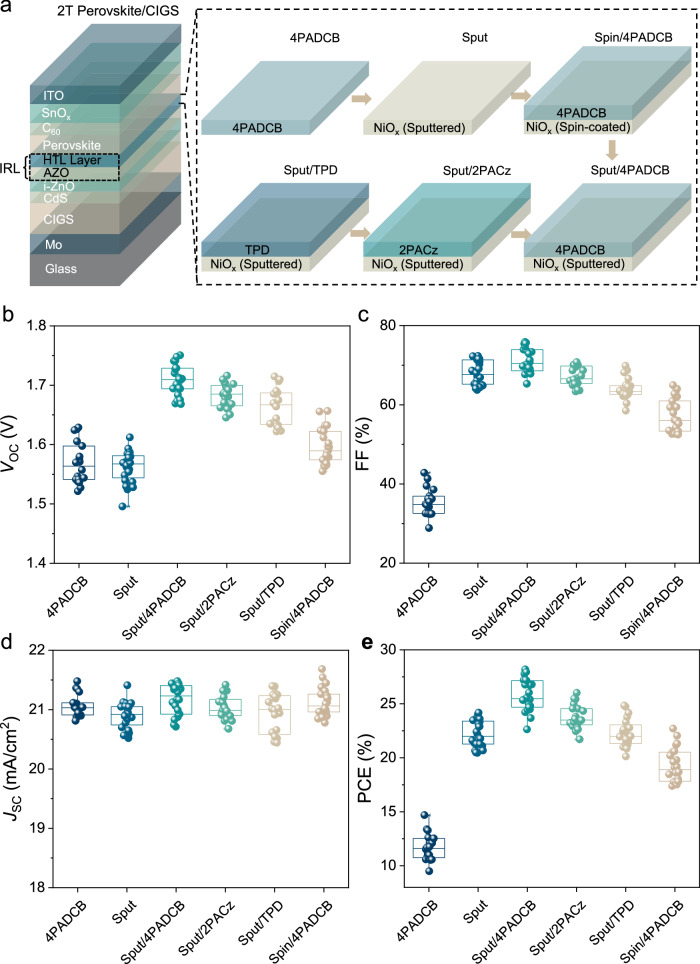
Performance of monolithic perovskite/CIGS tandem solar cells with different HTLs. **a** Schematic of the tandem solar cell structure incorporating different HTLs. **b–e** Comparison of key performance parameters: (**b**) *V*_OC_, (**c**) FF, (**d**) *J*_SC_, and (**e**) PCE for tandem cells with various HTLs indicated on the horizontal axis. Each data point corresponds to a statistical set (*n* ≥ 18). The open circle, top and bottom whiskers, and box indicate the mean, minimum and maximum values, and the 25%–75% interquartile range, respectively.

The advantages of using a NiO_x_/4PADCB double layer as a hybrid HTL for the perovskite top subcell were clearly demonstrated in Fig. 1. Through careful optimization of the CIGS fabrication process (Supplementary Figs. 1–8, Table 2), we successfully deposited the HTL directly onto the AZO transparent electrode surface of the as-grown CIGS subcell. X-ray photoelectron spectroscopy (XPS) results also confirmed that all three hole transport materials could be effectively deposited onto the sputtered NiO_x_ surface (Supplementary Fig. 9). When 4PADCB was deposited alone onto the AZO surface (4PADCB), the devices exhibited a very low FF (~35%) and a corresponding low PCE (~ 12%). Using sputtered NiO_x_ alone (Sput) significantly improved the FF to ~68%, as it effectively covered the surface of the CIGS subcell and eliminated microscale shunt paths. However, defects on the NiO_x_ surface limited the *V*_OC_ to a low level of ~1.57 V. By combining sputtered NiO_x_ with 4PADCB (Sput/4PADCB), surface defects were passivated, improving the FF to ~71% and boosting the *V*_OC_ to ~1.71 V. Replacing the sputtered NiO_x_ with spin-coated NiO_x_ (Spin/4PADCB) reduced both FF (~ 56%) and V_OC_ (~ 1.59 V), highlighting the superior ability of sputtered NiO_x_ to eliminate the leakage between the top and bottom subcells. As discussed in the introduction and detailed in Supplementary Table 1, the various IRLs used in prior perovskite/CIGS tandem studies have consistently suffered from either significant *V*_OC_ losses or low FF, or both^6,9,11,21^. These limitations often stemmed from incomplete interfacial defect passivation and inadequate shunt path removal. In contrast, our hybrid HTL with 4PADCB SAM on sputtered NiO_x_ successfully addressed these issues.

To maximize the benefits of the hybrid HTL, we carefully controlled the thickness and conductance of the sputtered NiO_x_ layer. As shown in Supplementary Fig. 10, the optimal thickness was determined to be ~20 nm. A thinner NiO_x_ layer failed to adequately cover the rough surface of CIGS subcells, leading to incomplete shunt path elimination and reduced FF and *V*_OC_. Conversely, a thicker NiO_x_ layer introduced increased internal resistance and carrier recombination, which also negatively impacted *V*_OC_. Conductivity of the NiO_x_ layer also played a critical role. As demonstrated in Supplementary Fig. 11, Table 3, incorporating oxygen into the sputtering atmosphere increased the Ni^3+^ density and the conductivity^28^, resulting in improved FF and *V*_OC_. However, excessive interstitial oxygen and Ni^3+^ ions would reduce the film quality and decrease FF. The optimal O_2_/(Ar+O_2_) pressure ratio was therefore established at 1%. As shown in Supplementary Fig. 12, both Grazing Incidence X-ray Diffraction (GI-XRD) and Raman analyzes indicate the formation of pure-phase nickel oxide phase without metallic nickel, with structural and conductivity changes correlating to oxygen content.

To evaluate the performance of 4PADCB against other organic hole transport materials, we combined sputtered NiO_x_ with two commonly used materials in single-junction perovskite solar cells: 2PACz and poly(N, N’-bis-4butylphenyl-N, N’-bisphenyl)benzidine (TPD)^29–31^ (Supplementary Fig. 13). As illustrated in Fig. 1, the devices exhibited a *V*_OC_ of ~1.69 V and an FF of ~67% for Sput/2PACz and ~1.66 V and ~63% for Sput/TPD. With comparable *J*_SC_ across all devices, the average PCEs incorporating TPD, 2PACz, and 4PADCB yielded 22.0%, 23.5%, and 25.5%, respectively, affirming 4PADCB as the superior choice for the hybrid HTLs. To understand these performance differences, both film thickness and molecular properties of the HTLs were considered. TPD usually forms a relatively thicker layer, potentially increasing internal resistance and reducing FF. In contrast, 4PADCB and 2PACz, as self-assembled monolayers, are much thinner and thus exert minimal influence on resistance. Beyond thickness, the advantages of 4PADCB are also linked to its distinct molecular structure, stronger interfacial dipole, and denser monolayer packing^15,32,33^.

Furthermore, we found that employing a thicker perovskite layer than is typically used in single-junction devices appreciably improved the performance of perovskite/CIGS tandems. In contrast to the double-pass light absorption that occurred in single-junction perovskite solar cells enabled by metallic electrode reflection, top subcells in tandems relied solely on single-pass absorption. As shown in Supplementary Figs. 14, 15, increasing the concentration of perovskite precursor solution would increase the average layer thickness, raising *J*_SC_ from ~20.6 mA/cm^2^ at 1.0 mmol/mL to ~21.6 mA/cm^2^ at 2.5 mmol/mL due to enhanced light absorption. Moreover, the thicker perovskite layer buried AZO protrusions more effectively, further mitigating microscale shunt paths and improving both *V*_OC_ and FF. Nevertheless, excessively increasing the perovskite thickness introduced tremendous non-uniformity, increasing internal resistance, carrier transport path, and recombination rate while limiting the isolation of AZO protrusions. These effects adversely affected *J*_SC_, *V*_OC,_ and FF. Balancing these factors, an optimal perovskite thickness of ~900 nm was determined (Supplementary Fig. 14), which aligns well with findings for perovskite/Si tandems.

### Interface quality and energy level alignment

**Fig. 2:**
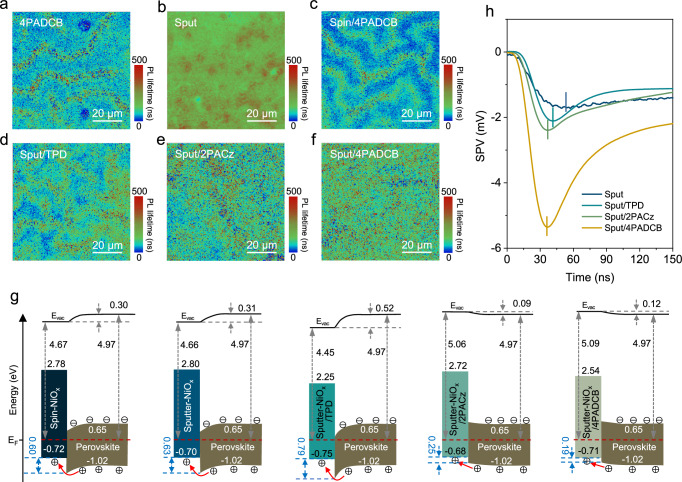
Charge-carrier dynamics and energy level alignment. **a–f** Photoluminescence lifetime images for perovskite films deposited on various HTLs: (**a**) 4PADCB, (**b**) Sput, (**c**) Spin/4PADCB, (**d**) Sput/TPD, (**e**) Sput/2PACz, and (**f**) Sput/4PADCB. **g** Energy level diagram illustrating the alignment between various HTLs and a wide-bandgap perovskite film. **h** Transient surface photovoltage for half-stack configurations of ITO/HTL/Perovskite/Au-Electrode with various HTLs.

To uncover the mechanisms behind the superior performance of the Sput/4PADCB hybrid HTL, we investigated the interface quality and energy level alignment using advanced characterization techniques. Confocal fluorescence lifetime imaging microscopy (FLIM) was employed to analyze the spatial distribution of photoluminescence (PL) lifetimes for perovskite films deposited on different HTLs. To eliminate interference from the rough CIGS surface, we used a smooth ITO substrate to form an ITO/HTL/perovskite stack. As shown in Fig. 2a, c, d, samples with 4PADCB, Spin/4PADCB, and Sput/TPD all showed relatively short PL lifetimes and considerable inhomogeneity. In contrast, samples Sput, Sput/2PACz, and Sput/4PADCB demonstrated enhanced PL lifetimes and notably improved uniformity (Fig. 2 b, e, f), with Sput/4PADCB performing the best. These results were further corroborated by surface potential measurements using Kelvin probe force microscopy (KPFM) (Supplementary Figs. 16, 17, Table 4) on different HTLs deposited on CIGS bottom cells under both ambient air and nitrogen environments. Large-area time-resolved PL (TRPL) measurements further quantified the improvement, with average carrier lifetimes of 15, 31, 74, 107, 161, and 217 ns, respectively, for Spin/4PADCB, 4PADCB, Sput/TPD, Sput, Sput/2PACz, and Sput/4PADCB (Supplementary Fig. 18, Table 5). Consistent trends were observed in TRPL measurements with excitation from both sides of the samples, as shown in Supplementary Fig. 19. The trend in steady-state PL intensity aligned with these findings, where higher PL intensities corresponded to longer carrier lifetimes. The Sput/4PADCB hybrid HTL demonstrated the most uniform interface, the longest radiative lifetime, and the highest PL intensity, confirming its superior ability to suppress non-radiative recombination at the perovskite/HTL interface.

To assess the hole extraction efficiency, we used ultraviolet photoelectron spectroscopy (UPS) to examine energy level alignment and transient surface photovoltage (tr-SPV) measurement to study the hole transport dynamics. As depicted in Supplementary Fig. 20, UPS data revealed the work functions and energy positions of the highest occupied molecular orbital (HOMO) or valence band maximum (VBM) for the various HTLs and perovskite layers. All HTLs displayed VBMs/HOMOs between 680 and 750 meV below the Fermi level, which were higher than that of the perovskite, enabling energetically possible hole extraction. As further demonstrated in Fig. 2g, in addition to a large valence band offset (~600, ~630, ~790 meV, respectively) for spin-coated NiO_x_, sputtered NiO_x_, and TPD-coated NiO_x_, the work function differences introduced an interface electric field between HTL and perovskite, resulting in a high energy barrier of ~300–520 meV. The large band offsets and energy barriers hindered hole transport from the perovskite layers to the HTLs, limiting extraction efficiency. In contrast, when 2PACz and 4PADCB were deposited on sputtered NiO_x_, their strong molecular dipole moment increased the work functions of the hybrid HTLs and reversed the interface electric field direction. The resulting small valence band offsets (~250 meV for 2PACz and ~190 meV for 4PADCB) and favorable electric fields facilitated efficient hole extraction.

The effectiveness of hole extraction for these interfaces was further validated by tr-SPV measurements. As shown in Fig. 2h, the tr-SPV signal from the ITO/HTL/perovskite/Au-Electrode half-stacks demonstrated about three-fold increase in amplitude, from −1.7 mV to −5.3 mV, accompanied by a reduction in extraction time from 53.3 ns to 36.0 ns in the sequence of Sput, Sput/TPD, Sput/2PACz, Sput/4PADCB (Supplementary Fig. 21a, Table 6). Similar trends were observed under nitrogen environments, with extraction times reducing from 67.3 ns to 23.3 ns (Supplementary Fig. 21b, Table 7). These results and the observed steep voltage rise for Sput/4PADCB indicated that the perovskite/4PADCB/NiO_x_ junction enabled the fastest and most efficient transport of photo-generated holes among all configurations tested. Collectively, the data indicated that 4PADCB provides multiple benefits when combined with sputtered NiO_x_: it passivates interfacial defects, minimizing carrier trapping and extending PL lifetimes, and it significantly improves energy level alignment, reducing transport barriers and enhancing hole extraction rates. These improvements translate directly to higher *V*_OC_ and FF for the tandem devices with sputtered NiO_x_/4PADCB hybrid HTL.

### Effect of ultrathin gold layer and performance of champion device

**Fig. 3:**
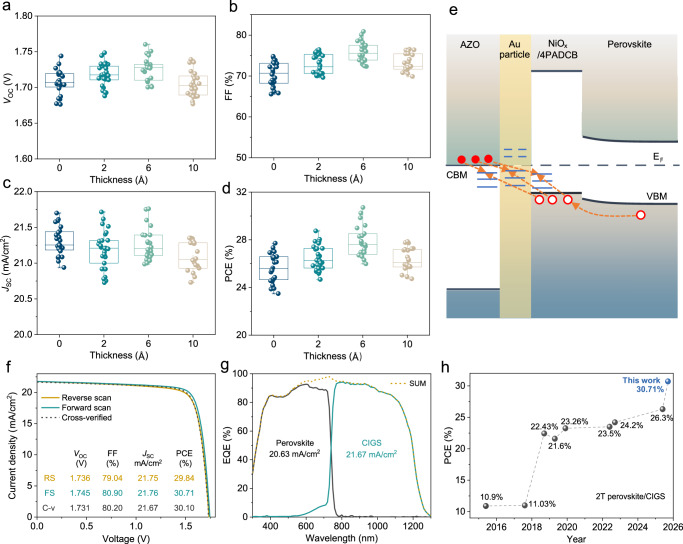
Performance of monolithic perovskite/CIGS tandem devices with the AZO/Au/NiO_x_/4PADCB IRL. **a–d** Comparison of *V*_OC_ (**a**), FF (**b**), *J*_SC_ (**c**), and PCE (**d**) for tandem devices with varying average Au layer thickness. **e** Schematic of the energy level diagram of the AZO/Au/NiO_x_/4PADCB recombination junction. **f** *J–V* curves for the champion tandem device. **g** External quantum efficiency (EQE) of the tandem device, where the slightly lower integrated *J*_SC_ may result from insufficient light intensity to saturate metastable defects. **h** Historical progress of record efficiencies for monolithic perovskite/CIGS tandem devices. Each data point corresponds to a statistical set (*n* ≥ 22). The open circle, top and bottom whiskers, and box indicate the mean, minimum and maximum values, and the 25%–75% interquartile range, respectively.

The proper IRL in a monolithic p-i-n (bottom)/p-i-n (top) tandem must consist of an electron transport layer (ETL) and an HTL that not only efficiently extracts electrons and holes from their respective light absorbers, but also recombines these carriers effectively and timely at the ETL/HTL junction^34^. For the optimized AZO/NiO_x_/4PADCB IRL, we introduced an ultrathin Au particle layer (Supplementary Fig. 22) at the AZO/NiO_x_ interface to further improve device performance. As shown in Fig. 3a–d, when the average Au thickness increased from 0 to 0.6 nm, the *J*_SC_ exhibited only a slight fluctuation, remaining around ~21.3 to ~21.2 mA/cm^2^, possibly due to minor light absorption by the Au particles. More importantly, the FF improved significantly from ~71% to ~76%. With the simultaneously increased *V*_OC_ from ~1.71 V to ~1.73 V, the average PCE boosted from 25.6% to 27.6%. When the average Au thickness exceeded 1 nm, FF saturated, and the overall device performance declined. These results highlight the critical role of the ultrathin Au particle layer in the AZO/Au/NiO_x_/4PADCB structure for enhancing FF and overall device performance.

To understand the role played by the thin Au particle layer, we plotted the energy level diagram of the AZO/Au/NiO_x_/4PADCB/perovskite stack in Fig. 3e. As shown in Supplementary Fig. 20, the VBM of sputtered NiO_x_ is close to the HOMO of 4PADCB and approximately 700 meV below the conduction band minimum (CBM) of the heavily doped AZO. Thus, the contribution from direct band-to-band tunneling to the carrier recombination between electrons from AZO and holes from NiO_x_ is negligible. This conclusion is supported by the resistive-like current-voltage (*J–V*) characteristics observed in the AZO/NiO_x_/4PADCB and AZO/Au/NiO_x_/4PADCB junctions (Supplementary Fig. 23). Without the Au particle layer, the carrier recombination was primarily mediated by interfacial trap states. Introducing the ultrathin Au particle layer opened up an additional recombination channel through Au particle states, and the insertion of an ultrathin Au layer could induce band bending at the Au/NiO_x_ interface due to the relatively high work function of Au, thereby facilitating hole extraction and increasing the recombination rate for majority carriers by ~12%, as evidenced by the increased current density of ~12% (Supplementary Fig. 23). This timely recombination of electrons and holes in IRL eliminated the accumulation of un-recombined charge carriers at the junction, reducing the reverse built-in electric field. The reduced charge carrier accumulation within the IRL due to the incorporation of the Au layer led to more efficient extraction of holes from the perovskite/4PADCB/NiO_x_ interface, thus reducing the residence time of holes and consequently the number of minority carrier recombination. A similar argument applies to electrons at the CIGS/CdS interface. In the end, a higher FF and *V*_OC_ for the tandem devices could be achieved, as evidenced by the improved *J–V* and dark *J–V* characteristics in Supplementary Fig. 24.

In addition to the Au particle layer, the AZO ETL layer was optimized to complete the ideal IRL. As shown in Supplementary Fig. 25, the optimal AZO thickness was determined to be ~200 nm. Thinner AZO significantly reduced both FF and *V*_OC_, likely due to ineffective electron collection, while thicker layers decreased *J*_SC_, likely due to parasitic absorption.

For the present tandem structure, incident light had to pass the C_60_ layer before reaching the perovskite layer. The absorptive nature of C_60_ in the ultraviolet (UV) region of the solar spectrum could also affect the performance of the top subcell. As demonstrated in Supplementary Figs. 26, 27 for single semi-transparent perovskite solar cells, a normal thickness of C_60_ at 20 nm indeed reduced the *J*_SC_ by incidence light from the top TCO side, and a reduced thickness at ~12 nm could reach a better overall performance. As further illustrated in Supplementary Fig. 28, an optimal thickness of ~12 nm, in contrast to ~20 nm normally used for stand-alone opaque perovskite solar cells, could effectively avoid strong UV parasitic light absorption and maintain reasonable *V*_OC_ and FF for the tandem devices.

Using the optimized composite IRL of AZO/Au/NiO_x_/4PADCB, along with previously optimized bottom and top subcells (Supplementary Fig. 29)^35^, we fabricated a champion cell with a 0.15 cm^2^ active area. The device achieved a PCE of 30.71%, with a *V*_OC_ of ~1.745 V, an FF of ~80.9%, and a *J*_SC_ of ~21.76 mA/cm^2^ (Fig. 3f, corresponding EQE spectra shown in Fig. 3g). The PCE cross-verified by an external organization was 30.1%, with a *V*_OC_ ~ 1.731 V, an FF ~ 80.2%, and a *J*_SC_ ~ 21.67 mA/cm^2^ (Supplementary Note 1). Additionally, a larger area of 0.51 cm^2^ tandem device was fabricated, achieving an efficiency of 28.04% (Supplementary Fig. 30). As shown in Fig. 3h, this work marks a significant milestone in the development of monolithic perovskite/CIGS tandem solar cells. Our device not only sets a notably high efficiency for monolithic perovskite/CIGS tandems but also surpasses the performance of four-terminal architectures (Supplementary Table 8). Furthermore, it also exceeds the record efficiencies of both constituent single-junction solar cells in their optimized configurations (perovskite: 27.0%, CIGS: 23.6%, NREL chart, 2025)^16^. Compared to the previous champion perovskite/CIGS tandem devices (Supplementary Table 1), the significantly improved PCE can be attributed primarily to the remarkable enhancement in both FF and *J*_SC_. The moderate *V*_OC_ resulted from deliberate bandgap optimization, with the CIGS bottom subcell set at 1.01 eV and the perovskite top subcell at 1.67 eV, enabling efficient current matching. The exceptional FF value exceeding 80%, one of the highest reported for perovskite/CIGS tandems^22^, surpassed that of standalone CIGS subcells, demonstrating the effectiveness of the optimized IRL. The high *J*_SC_ was achieved through the use of a narrow-bandgap CIGS bottom subcell, a thicker perovskite absorber, optimized bandgap matching, and an anti-reflection coating.

While 4-terminal (4 T) perovskite/CIGS tandems have shown high efficiency^7,23^ and favorable energy yield under real-world conditions, detailed modeling has demonstrated that 4 T configurations deliver only a modest increase in annual energy yield compared to their 2-terminal (2 T) counterparts, typically about 1–3%, depending on deployment location and tracking strategy^36^. In earlier studies, the monolithic 2 T devices have so far exhibited lower performance, their limitations primarily stem from non-optimized interfacial recombination layers rather than intrinsic material or structural constraints. Here, we demonstrate a monolithic perovskite/CIGS tandem with the PCE exceeding 30% through the introduction of a multifunctional AZO/Au/NiO_x_/4PADCB interlayer, highlighting its potential for scalable, high-yield photovoltaic applications.

### Stability of perovskite films and tandem devices

**Fig. 4:**
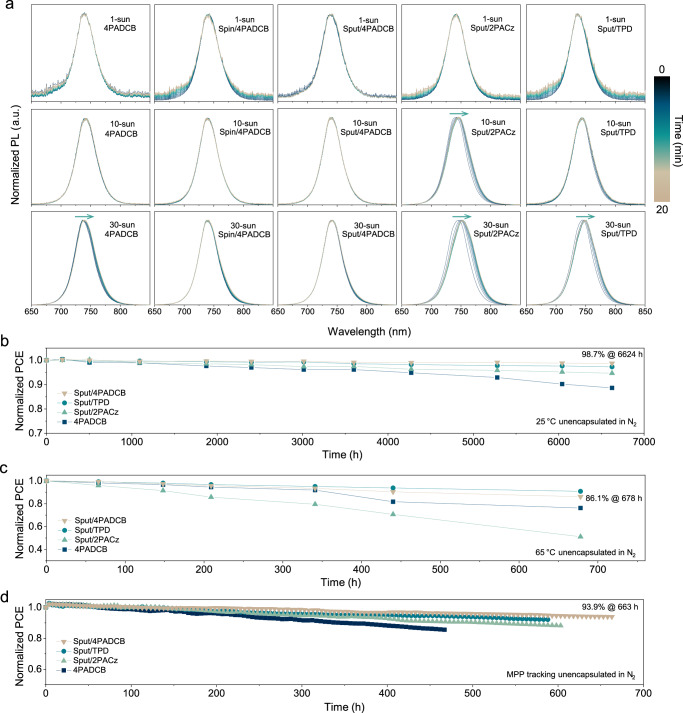
Photostability of perovskite films and thermal stability of tandem devices. **a** Normalized evolution of PL spectra for perovskite films on different HTLs (4PADCB, Spin/4PADCB, Sput/TPD, Sput/2PACz, and Sput/4PADCB) under equivalent 1-sun, 10-sun, and 30-sun illumination over 20 min. **b** Long-term storage stability of monolithic perovskite/CIGS tandem devices at room temperature (25 °C) in an N_2_ atmosphere. **c** Thermal stability of tandem devices at 65 °C in an N_2_ atmosphere. **d** Continuous MPPT test for unencapsulated tandem devices. Note that absolute efficiency data for Figures (**b**–**d**) are provided in Supplementary Fig. 33, and these measurements were all performed under standard 1-sun illumination conditions.

We evaluated the photostability of perovskite films with various hole transport layers in ambient conditions under continuous-wave laser illumination at different light intensities. Time-dependent and excitation light intensity-dependent PL measurements were conducted at 405 nm wavelength. As shown in Fig. 4a, at 1-sun-equivalent intensity, all five perovskite films exhibited minimal changes in their PL spectra, indicating good photostability under standard operating conditions. To accelerate or amplify the light-induced phase segregation process, the illumination intensity was further increased to 10- and 30-sun equivalents, aiming to elucidate how different interfacial structures affect device stability. Notably, only the Spin/4PADCB and Sput/4PADCB samples maintained nearly unchanged PL spectra even under such high-intensity illumination. Supplementary Fig. 31 presents the PL spectra normalized to the maximum value within each data set (i.e., from 0 to 20 min), which facilitates the observation of intensity changes over time. Considering both PL intensity and spectral shifts, 4PADCB appears to exhibit relatively better stability compared to 2PACz and TPD. Light-induced halide segregation in wide-bandgap perovskites with high Br/I ratios is often linked to PL instability due to the formation of iodide-rich clusters. While charge accumulation has been proposed to accelerate this process by promoting ion migration^32,37,38^, the segregation mechanism is likely multifactorial and still requires further clarification.

Due to their ultrathin nature, SAMs can rapidly transport photo-generated holes, effectively suppressing light-induced phase segregation^10,32,38^. Although 4PADCB and 2PACz share identical anchoring groups and exhibit similar defect passivation on sputtered NiO_x_ surfaces, they differ in their functional groups: 4PADCB possesses a 7H-dibenzo[c,g]carbazole, while 2PACz contains a carbazole. The functional group in 4PADCB features expanded π-orbitals arranged in a non-coplanar helical configuration. This structure enables denser and more ordered molecular packing via steric repulsion, resulting in better spatial overlap between the expanded π-orbitals and the perovskite wavefunction^15^. As demonstrated in Fig. 2h, this characteristic facilitates more efficient hole extraction, reducing charge accumulation and thereby suppressing light-induced phase segregation. In contrast, 2PACz, with its carbazole functional group, exhibited inferior photo-stability due to its relatively less effective molecular packing and hole extraction properties. As a conventional hole transport material, TPD offers reasonable energy level alignment and hydrophobicity^39^ but often forms non-uniform films on rough surfaces like CIGS^40^. In contrast, SAMs spontaneously form conformal, ultrathin layers, ensuring better surface coverage on textured substrates and thereby improving interfacial contact and reducing trap states. As a result, SAM-based devices exhibit enhanced performance. Supplementary Figs. 13, 32 present the molecular structures^15,32,33^, Quasi-Fermi Level Splitting (QFLS) analysis and pseudo *J–V* of the three HTLs obtained from the intensity-dependent *V*_OC_, confirming that 4PADCB significantly mitigates *V*_OC_ loss and boosts device efficiency, supporting the use of NiO_x_/4PADCB as an effective hybrid hole transport layer.

We further assessed the thermal stability of unencapsulated tandem devices stored in a nitrogen environment at different temperatures. After 6600 h of storage at 25 °C (Fig. 4b), the 4PADCB device retained 88.6% of its initial efficiency. In contrast, the Sput/4PADCB device demonstrated significantly improved stability, maintaining 98.7% of its initial efficiency. At 65 °C, after 678 h (Fig. 4c), the Sput/4PADCB device retained about 86% of its initial efficiency, slightly below the Sput/TPD device but significantly outperforming both 4PADCB and Sput/2PACz devices.

Maximum power point tracking (MPPT) measurements further reinforced these findings. The Sput/4PADCB device exhibited the highest stability, while the 4PADCB device showed the greatest degradation (Fig. 4d). The enhanced stability of NiO_x_-containing devices is likely attributed to the abundant surface hydroxyl (OH) groups on NiO_x_, which facilitate stronger molecular anchoring of SAM layers^41^. The superior stability of 4PADCB SAM over 2PACz SAM results from its ability to form denser and more uniform molecular packing.

In conclusion, the optimized AZO/Au/NiO_x_/4PADCB IRL delivered exceptional PCE for perovskite/CIGS tandems. The combination of sputtered NiO_x_ and 4PADCB effectively eliminated the microscale shunt paths and efficiently passivated the interfacial traps. The ultrathin Au particle layer substantially boosted the recombination rate of the majority carriers from subcells. The efficient charge transport, coupled with strong molecular anchoring on NiO_x_, enabled the perovskite/CIGS tandem devices to maintain robust long-term performance under elevated temperatures and prolonged light exposure. The principles behind this composite IRL design can be extended to other tandem architectures, offering a pathway toward the development of high-efficiency, stable, and durable multi-junction solar cells.

## Methods

### Materials

Formamidinium iodide (FAI), Cesium iodide (CsI), Lead iodide (PbI_2_), Lead bromide (PbBr_2_), Lead chloride (PbCl_2_), Piperazinium Diiodide (PDI), Piperazine hydrobromide (PBr) and C_60_ were obtained from Xi’an Polymer Light Technology in China. Dimethyl sulfoxide (DMSO), N, N-dimethylformamide (DMF), ethanol, and isopropanol (IPA) were purchased from Sigma-Aldrich. Diethyl ether (DE) was purchased from Jiangsu Yonghua in China. Poly(N, N’-bis(4-butylphenyl)-N, N-bis(phenyl)benzidine) (poly-TPD) was purchased from 1-Materials in Canada. 2PACz ([2-(9H-carbazol-9-yl)ethyl]phosphonic acid) was purchased from TCI. 4PADCB was synthesized by our team. Tetrakis(dimethylamino)tin(IV) (99.9999%) for atomic-layer-deposited (ALD) SnO_2_ was bought from Suzhou Xinjiayuan Chemical Technology Co., Ltd. Indium tin oxide (ITO) and NiO_x_ targets were purchased from Shenzhen Zhongchengda Applied Materials Co., Ltd. Au(99.999%) was purchased from Hebei Fengming New Material Technology Co., Ltd. NiO_x_ nanoparticle powder (particle size around 10 nm) was purchased from Advanced Election Technology Company in China. ITO/glass substrates were purchased from Yiyang Huanan Xiangcheng Technology Co., Ltd, with a sheet resistance of 15 Ω/sq. Cu, In, Ga, and Se pellets were supplied by Kurt J. Lesker Company. Mo, intrinsic ZnO, and Al-doped ZnO targets were supplied by Huizhou Tianyi Rare Materials Co., Ltd.

### Fabrication of single-junction wide-bandgap perovskite solar cells

The perovskite solar cell was fabricated in a p-i-n structure with ITO/4PADCB/perovskite/C60/BCP/Ag. First, a 4PADCB solution (0.5 mg/ml in ethanol) was spin-coated directly onto an ITO substrate at 3000 rpm for 30 s, followed by annealing at 100 °C for 10 min. Then, the 4PADCB-coated ITO sample was pre-washed by spin-coating a PBr solution (1.0 mg/ml) at a speed of 5000 rpm (with a ramping rate of 3000 rpm/s) for 30 s. Wide-bandgap perovskite solutions were prepared by dissolving FAI, CsI, PbI_2_, PbBr_2_, PbCl_2_, with molar ratios adjusted to form (FA_0.8_Cs_0.2_)Pb(I_0.82_Br_0.15_Cl_0.03_)_3_ in a DMF and DMSO mixed solvent system (DMF: DMSO = 3:1 volume ratio). This solution was spin-coated onto the PBr film at 3000 rpm for 60 se. During this process, 500 μl of DE was quickly dripped onto the center of the perovskite film 30 s before the end of the spin-coating process, followed by annealing at 100 °C for 20 min. The surface passivation of the perovskite film was achieved by spin-coating a PDI solution (0.5 mg/ml). For the opaque devices, C_60_ (20 nm), BCP (6 nm), and Ag (100 nm) layers were sequentially deposited onto the perovskite films by thermal evaporation.

### Fabrication and characterization of single-junction CIGS solar cell

The detailed CIGS and CdS preparation procedures are provided in Supplementary Figs. 1–4. Briefly, CIGS fabrication began with sputtering a ~1 μm molybdenum layer onto soda-lime glass as the back electrode. Then, Cu, In, Ga, and Se were co-evaporated via the multi-stage process to form a CIGS light absorber layer (~2.7 μm). The RbF post-deposition treatment was performed to improve the junction quality at the CIGS surface. The CIGS devices were completed with a ~50 nm CdS buffer layer via chemical bath deposition, a ∼50 nm i-ZnO layer, and a ~ 200 nm (for tandem devices) Al-doped ZnO window layer via RF sputtering. In the stand-alone configuration, Al grids and MgF_2_ antireflection layers were additionally deposited by thermal evaporation.

### Fabrication of perovskite/CIGS monolithic tandem solar cells

For the fabrication of two-terminal tandem devices, the CIGS cells were terminated at the AZO layer. In the optimized scheme, a thin layer of 0.6 nm Au was deposited by thermal evaporation under high vacuum conditions (5 × 10^−4^ Pa). In other schemes, this step was skipped. Immediately afterward, 20 nm of NiO_x_ was sputtered in an O_2_ (1%)/Ar (99%) mixed atmosphere at a process pressure of 0.5 Pa and a sputtering power of 60 W. An organic hole transport layer of either TPD, 2PACz, or 4PADCB was further spin-coated on the NiO_x_ layer. The light absorber layer of wide-bandgap perovskite was deposited in the same way as described above for the single-junction perovskite solar cells. It is worth noting that the concentration of the perovskite solution was now 1.80 mmol/mL, rather than the 1.35 mmol/mL concentration used for single-junction perovskite solar cells. To decrease parasitic absorption, a thinner C_60_ layer (12 nm) was deposited by thermal evaporation, and a 20 nm thick SnO_x_ layer, instead of BCP, was deposited by reactive atomic layer deposition (ALD) (Picosun R200). The pulse and purge times were 0.3 s and 12 s, respectively, for TDMASn and 4 s and 15 s, for H_2_O. The front transparent electrode of 100 nm thick ITO (In/Sn ratio 90/10) was sputtered in a pure argon atmosphere, and the frame electrode of 300 nm thick Ag was deposited by thermal evaporation. Finally, the MgF_2_ antireflection layer (~100 nm) was deposited using electron beam evaporation.

### Device characterization

*J–V* curves were measured using an AM 1.5 G solar simulator (Enli-tech AAA solar simulator, China) under ambient conditions with a scan speed of 0.1 V s^−1^ (voltage steps of 20 mV and a delay time of 30 ms). The voltage range was from −0.1 V to 1.25 V for single-junction devices and −0.1 V to 2.0 V for tandem devices. The intensity of the solar simulator was calibrated to 1 sun (100 mW cm^−2^) using a reference Si solar cell. The boxes in the PV parameter plots indicate the 25/75 percentiles, and the whiskers mark the 10/90 percentiles. The line in the plots marks the respective average value.

The EQE curves were measured using a QE/incident photon-to-electron conversion efficiency (IPCE) system (Enli Technology Co. Ltd., China). The system was calibrated using a Si reference cell for the range from 300 to 1100 nm and a Ge reference cell for the range from 1100 to 1400 nm. For the tandem devices, LED bars were used as bias light sources, illuminating at a wavelength of 850 nm to measure the top cell and at a wavelength of 550 nm to measure the bottom cell.

### Morphology characterization

The morphology and microstructure of the various samples were characterized using field-emission scanning electron microscopy (SEM, TESCAN MIRA3). Except for the Au particle on the conductive AZO sample, the remaining samples were coated with a thin gold layer (~5 nm, deposited for 30 s at 30 mA) to mitigate charging effects. SEM imaging was conducted under high vacuum, utilizing a beam energy of 15 kV.

### Grazing Incidence X-Ray Diffraction (GI-XRD)

GI-XRD analysis was carried out using a Rigaku SmartLab 3 kW diffractometer (Rigaku Co., Japan) with Cu K*α* radiation, operated at a scanning speed of 5° min^−^¹.

### Raman spectrum measurement

The Raman spectrums were collected by a CCD detector (PIXIS256) through a spectrometer (Princeton Instrument, SpectraPro HRS-300) with a 1200 grooves/mm grating for a resolution of less than 1 cm^−1^. The slit was fixed to 100 μm for 1 mi integration. A 532 nm laser was used to excite the samples through a 100$\times$ objective lens.

### Ultraviolet photoelectron spectroscopy (UPS) measurements and X-ray photoelectron spectroscopy (XPS) measurements

UPS measurements were performed on a Thermo Fisher ESCALAB Xi^+^ instrument, and the samples were placed in an ultrahigh vacuum. The measurements were conducted with an ultraviolet light source of He I (21.2 eV) with a pass energy of 0.5 eV, dwell time of 50 ms, bias voltage of −6 V, and a step size of 20 meV. The systematic energy error of UPS was ~50 meV. The relative accuracy of the work function and valence band maximum position was slightly better than 50 meV. UPS is very sensitive to the cleanliness of the film surface. To avoid contamination errors, we kept the freshly prepared samples in a nitrogen-filled box until they entered the UPS chambers. XPS measurements were performed on a Thermo Fisher ESCALAB Xi^+^ instrument with a monochromatic Al Kα (1486.6 eV) X-ray, and the samples were measured under an ultrahigh vacuum. The binding energies were calibrated using the C(1 *s*) carbon peak (284.8 eV). All the high-resolution spectra were obtained under constant analyzer energy mode with a pass energy of 30 eV and a step size of 0.1 eV.

### Atomic force microscopy (AFM) and Kelvin probe force microscopy (KPFM)

AFM height images and KPFM surface potential distribution images were obtained under ambient conditions using a Bruker Dimension Icon AFM. These measurements employed amplitude modulation mode, using SCM-PIT-V2 probes from Bruker, which have a resonance frequency of 70 kHz and a force constant of 3 N/m. A consistent lift height of 20 nm was maintained throughout the measurements. Regarding the measurement under nitrogen atmosphere, we maintain the same testing conditions and use a custom-built acrylic box with continuous nitrogen purging to prevent ambient degradation for KPFM measurements.

KPFM measurements directly provided the spatially resolved contact potential difference (CPD), which was defined as

1 $${V}_{{{\rm{CPD}}}}=\left({\varPhi }_{{{\rm{sample}}}}-{\varPhi }_{{{\rm{tip}}}}\right)/e$$

where *Φ*_tip_ and *Φ*_sample_ were the work functions of the tip and sample, respectively, and *e* was the elementary charge. When a standard reference, such as highly oriented pyrolytic graphite (HOPG) with a known work function, was used, the absolute work function of samples could be determined by

2 $${\varPhi }_{{{\rm{sample}}}}={\varPhi }_{{{\rm{HOPG}}}}+e\left[{V}_{{{\rm{CPD}}}}\left({{\rm{sample}}}\right)-{V}_{{{\rm{CPD}}}}\left({{\rm{HOPG}}}\right)\right]$$

### Photoluminescence (PL) and time-resolved photoluminescence (TRPL) measurements

The PL spectra of the perovskite films were recorded using a DeltaFlex fluorescence spectrometer (HORIBA, Japan), excited with a semiconductor laser at a center wavelength of 488 nm. TRPL was carried out with a time-correlated single-photon counting (TCSPC) module as the detector. Carrier lifetime is measured using TRPL decay curves. The fitting parameters, obtained from a bi-exponential decay equation, are listed below:

3 $${\tau }_{{ave}}=({A}_{1}{\tau }_{1}^{2}+{A}_{2}{\tau }_{2}^{2})/({A}_{1}{\tau }_{1}+{A}_{2}{\tau }_{2})$$

where ${\tau }_{{{\rm{ave}}}}$ is the average carrier lifetime, ${A}_{1}$ and ${A}_{2}$ represent the decay amplitudes of fast and slow decay processes, respectively, and ${\tau }_{1}$ and ${\tau }_{2}$ stand for the fast and slow decay time constants, respectively.

### Fluorescence lifetime imaging microscopy (FLIM)

The FLIM measurement was performed with the confocal configuration of a PicoQuant MicroTime 200 instrument. The excitation pulsed laser at 488 nm was operated at a repetition rate of 5 MHz and was focused onto the sample through an objective lens (Olympus MPLFLN100x, NA 0.9). The fluorescence emission of the sample was passed through a 650 nm long-pass filter (Jcoptix, OFE1LP-650) and detected by a single-photon avalanche diode (SPAD). The electrical signal was then processed by time-correlated single-photon counting (TCSPC) electronics (Time Harp 260, PicoQuant). The FLIM images were analyzed by PicoQuant SynphoTime 64.

### Hall measurement

The Hall measurements were characterized by the Hall effect tester (HMS-5500, Ecopia). Place the sample in the testing module at room temperature and atmospheric conditions to obtain information on the sheet resistance, resistivity, and carrier concentration of the thin film.

### Photoluminescence (PL) photo-stability test and Photoluminescence quantum yield (PLQY) test

Photostability measurements were performed using a home-built setup. The perovskite films were excited by a 405 nm continuous-wave laser, and the illumination intensity was adjusted to 1-, 10-, and 30-sun-equivalent intensity using a variable neutral density filter (LBTEK, NDFR-50S-3M). Each measurement lasted a total of 20 min, with the fluorescence spectrum collected every minute to characterize the sample’s stability under illumination. The PLQY measurements were characterized by the luminescence test system (Enli Technology Co. Ltd, China, LQ-100), with an excitation wavelength of 365 nm.

### Transient surface photo-voltage (tr-SPV) measurement

Measurements were conducted at 10 Hz in an ambient environment using a 405 nm nanosecond pulsed diode laser (Thorlabs, NPL41C) with a pulse duration of 6 ns and an intensity of approximately 0.75 μJ/cm^2^, triggered by an arbitrary function generator (Tektronix, AFG31000). The tr-SPV signal was measured using an oscilloscope (Tektronix, MDO34, 2.5 GS/s). The measurement frequency was 10 Hz, and 128 averages were taken per routine. Meanwhile, to avoid atmospheric impact, we conducted SPV measurements under nitrogen atmosphere using a custom-built acrylic box with continuous nitrogen purging to prevent ambient degradation, while ensuring that other testing conditions were the same. The sample was excited from the ITO side, and the SPV signals were collected using two copper wires, which connected the Au electrode on the front side and the ITO electrode on the back side of the half device. These open-circuit signals were then transferred to the oscilloscope through a BNC adapter. Carrier extraction time is measured using tr-SPV curves. It is defined as the time required to reach the maximum SPV. The decay time is determined by fitting the data to a bi-exponential decay equation, as described below:

4 $$V\left({{\rm{t}}}\right)={V}_{10}\exp \left(-t/{\tau }_{1}\right)+{V}_{20}\exp \left(-t/{\tau }_{2}\right)$$

where ${V}_{10}$ and ${V}_{20}$ represent the decay amplitudes of fast and slow decay processes, respectively, and ${\tau }_{1}$ and ${\tau }_{2}$ stand for the fast and slow decay time constants, respectively.

### Reporting summary

Further information on research design is available in the Nature Portfolio Reporting Summary linked to this article.

## Supplementary information

Supplementary Information

Solar Cell Reporting Summary

Transparent Peer Review file

## Source data

Source data

## Data Availability

The main data supporting the findings of this study are available within the published article and its Supplementary Information and source data files. Additional data are available from the corresponding author on request. Source data are provided with this paper.
