# Supplementary material for: Efficient perovskite/Cu(In,Ga)Se2 tandem solar cells with a composite intermediate recombination layer
Source: Nat Commun. 2025 Dec 10;17:711. doi: 10.1038/s41467-025-67350-y (PMC12820335; doi:10.1038/s41467-025-67350-y)
Supplement: Supplementary file 1 — Supplementary Information [file 41467_2025_67350_MOESM1_ESM.pdf]

Supplementary Information for

# Efficient Perovskite/$Cu(In,Ga)Se_2$ Tandem Solar Cells with a Composite Intermediate Recombination Layer

Wang Li[1]†, Junjun Zhang[1]†, Li Zeng[1]†, Wanhai Wang[2], Zhou Fang[1], Xinxing Liu[1], Zengyang Ma[1], Yitian Zhang[1], Hui Yan[1], Chen Shen[1], Zhuo Xue[1], Jingyi Zhu[1], Qiren Luo[1], Chang Liu[1], Ruixuan Jiang[3], Tongle Bu[3], Weihua Tang[2], Jianmin Li[1*], Sheng Wang[1*], Junbo Gong[1*], Xudong Xiao[1*]

[1] School of Physics and Technology, Key Lab of Artificial Micro- and Nano-structures of Ministry of Education, Wuhan University, Wuhan 430072, Hubei, China

[2] Institute of Flexible Electronics (IFE Future Technologies), College of Materials, Innovation Laboratory for Sciences and Technologies of Energy Materials of Fujian Province (IKKEM), Xiamen University, Xiamen 361005, China

[3] State Key Laboratory of Advanced Technology for Materials Synthesis and Processing, Wuhan University of Technology, Wuhan 430070, PR China.

* Corresponding author. Email: ljmphy@whu.edu.cn (J.M.L.); shengwang16@whu.edu.cn (S.W.); gongjunbo@whu.edu.cn (J.B.G.); xdxiao@whu.edu.cn (X.D.X.)

† These authors contributed equally to this work

**Table of contents**

Supplementary Figures 1-33

Supplementary Tables 1-8

Supplementary Note 1

Supplementary references

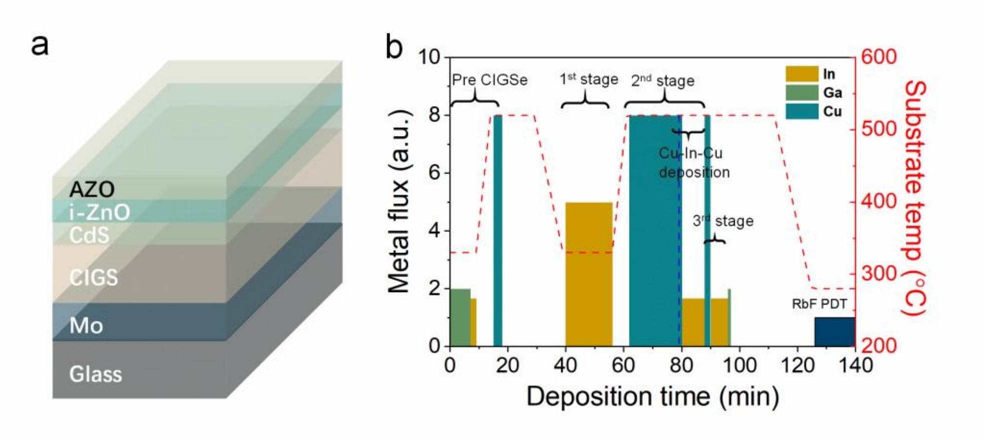


**Supplementary Fig. 1 |** Schematic diagram of the CIGS solar cell structure (**a**) and the growth sequence profile (**b**).

The fabrication details of the narrow-bandgap CIGS subcell are described in Ref. 1. As shown in Supplementary Fig. 1a, the CIGS bottom subcell in the tandem structure adopts a glass/Mo/CIGS/CdS/i-ZnO/AZO configuration. Consistent with our previous report (Supplementary Fig. 1b), the CIGS absorber was deposited on Mo-coated soda-lime glass, where elevated processing temperatures enabled sodium diffusion from the substrate into the CIGS layer via the molybdenum back contact. First a pre-CIGS layer with high Ga content was introduced at a high temperature of 520℃ to suppress the Ga diffusion and enhance back Ga grading. Subsequently, the substrate temperature was lowered to 330℃, followed by a 20-minute evaporation of In alone. In the second stage, after reaching the stoichiometric point, an additional 15% Cu excess was deposited. Finally, in the third stage, the surface Ga content was approximately 10% of the Ga content in the first stage. After completing the growth of the CIGS absorber layer, a post-deposition treatment (PDT) with RbF was performed without breaking the vacuum. The RbF was deposited at a substrate temperature of 280℃ and a source temperature of 400℃ for 20 minutes, resulting in an RbF film with a thickness of approximately 20 nm. Following the deposition of the CIGS absorber layer, a ~50 nm CdS buffer layer was deposited using the chemical bath deposition (CBD) method. Subsequently, a 50 nm intrinsic ZnO layer and a 250 nm AZO layer were deposited by RF sputtering.

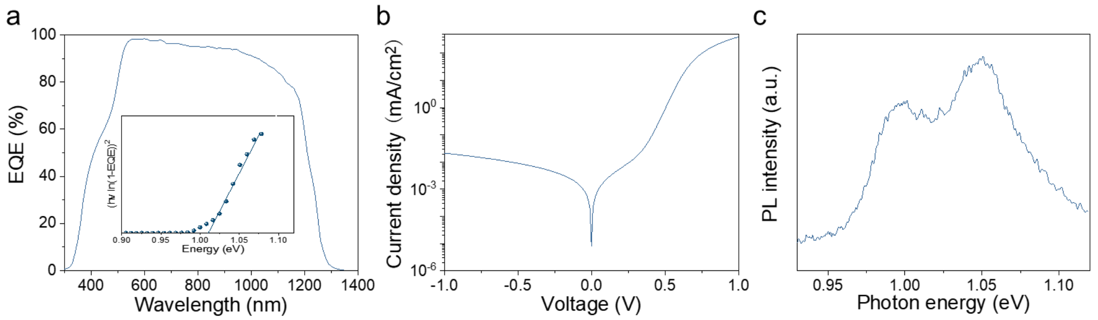


**Supplementary Fig. 2 |** Characterization of CIGS. EQE curves of CIGS device (**a**), Dark *J*-*V* curve of CIGS device (**b**), and PL spectrum of CIGS layer (**c**).

As shown in Supplementary Fig. 2a, the EQE curve of the CIGS subcell under optimal conditions indicates a bandgap of approximately 1.01 eV and a high integrated photocurrent. The dark *J*-*V* curve in Supplementary Fig. 2b further reveals low recombination losses, consistent with our previous findings[1], where more detailed device characteristics are discussed. In Supplementary Fig. 2c, the PL spectrum shows a dominant peak corresponding to a bandgap of ~1.01 eV, confirming the absorber's quality, while the secondary peak may originate from phases with higher Ga content in the absorber.

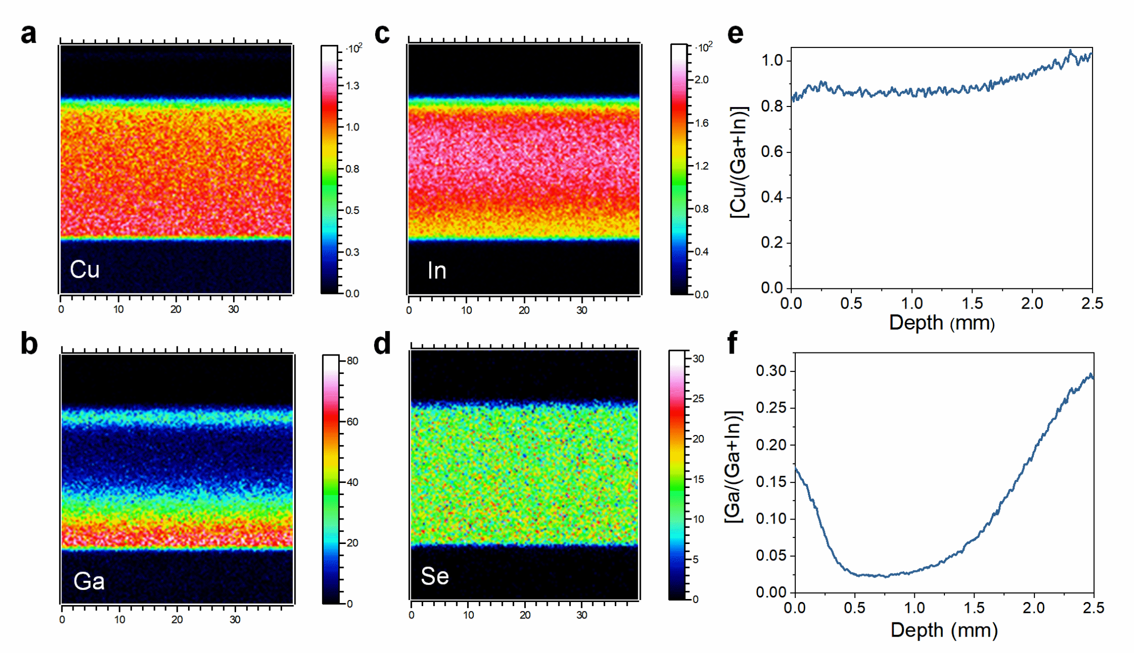


**Supplementary Fig. 3 |** Characterizations of CIGS by ToF-SIMS. **a-d**, ToF-SIMS element mapping of Cu (**a**), Ga (**b**), In (**c**), and Se (**d**). **e-f**, Depth profiles of CGI (**e**) and GGI (**f**) calculated from ToF-SIMS data.

Supplementary Fig. 3a-d clearly show the distribution of various elements both on the surface and inside the CIGS absorber layer by the SIMS images, while the CGI and GGI in Supplementary Fig. 3e-f, reflecting compositional ratios at different depths, further helps in understanding the composition of CIGS cells.

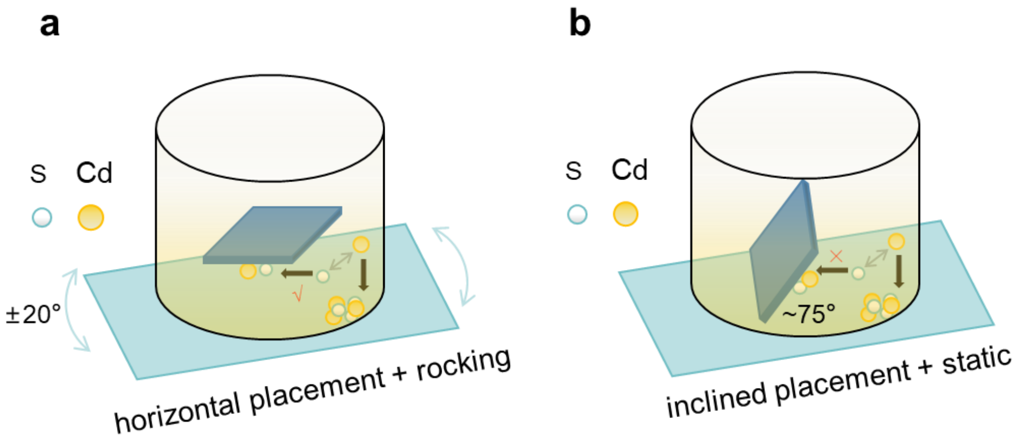


**Supplementary Fig. 4 |** Schematic diagram of chemical bath deposition under (**a**) dynamic (rocking) and (**b**) static conditions.

The CdS films were deposited using a chemical bath deposition (CBD) process. Aqueous precursor solutions consisting of 40 mL of 1.5 mM cadmium sulfate ($CdSO_4$), 26 mL of ammonia solution (26-28% concentration), and 134 mL of 75 mM thiourea ($CH_4N_2S$) were sequentially added into a clean glass beaker containing the sample, resulting in a total volume of 200 mL. The beaker was then placed in a water bath maintained at 65 °C. Deposition was performed either under rocking at 30 cycles per minute (cpm) for 15 minutes or under static conditions for 17 minutes. After deposition, the sample was immediately rinsed with deionized water, dried with $N_2$, and annealed at 150 °C for 2 minutes.

As illustrated in Supplementary Fig. 4a, during rocking-assisted deposition, the sample was held horizontally with the rocking amplitude is approximately ±20°. In contrast, for static deposition, the sample was inclined at approximately 75° from the horizontal (Supplementary Fig. 4b) without stirring. This difference in deposition mode, including both sample orientation and the rocking, has a pronounced impact on the resulting film uniformity and surface roughness.

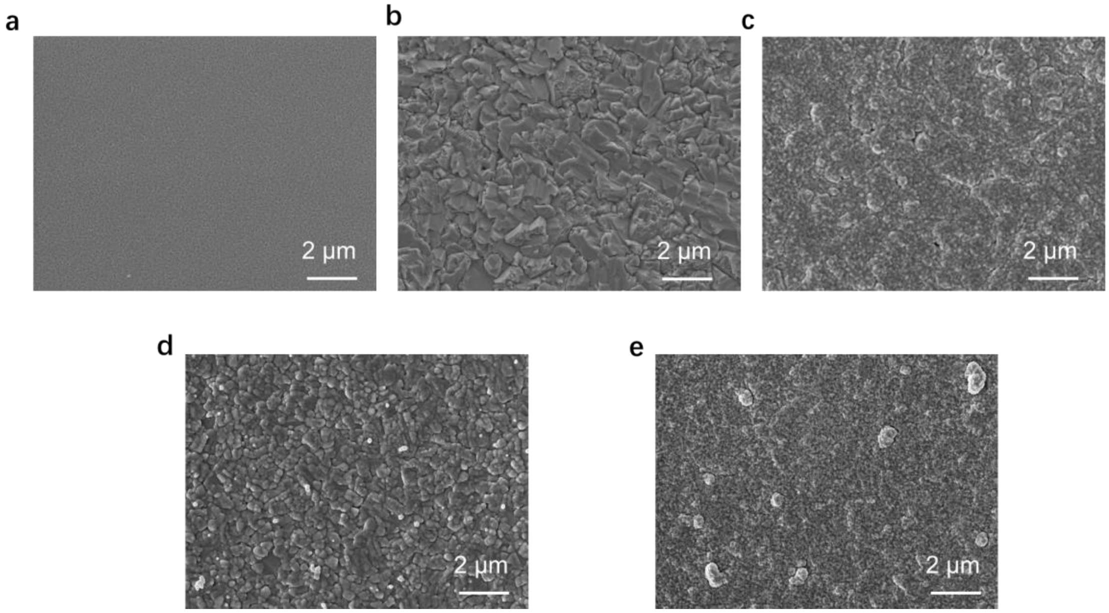


**Supplementary Fig. 5 |** SEM images of different surfaces. AZO on glass (**a**), Bare CIGS (**b**), AZO on CIGS (**c**), CdS on CIGS (**d**), AZO on CdS/CIGS (**e**). These images demonstrated that the large surface particles originated from the CdS deposition process rather than the AZO deposition.

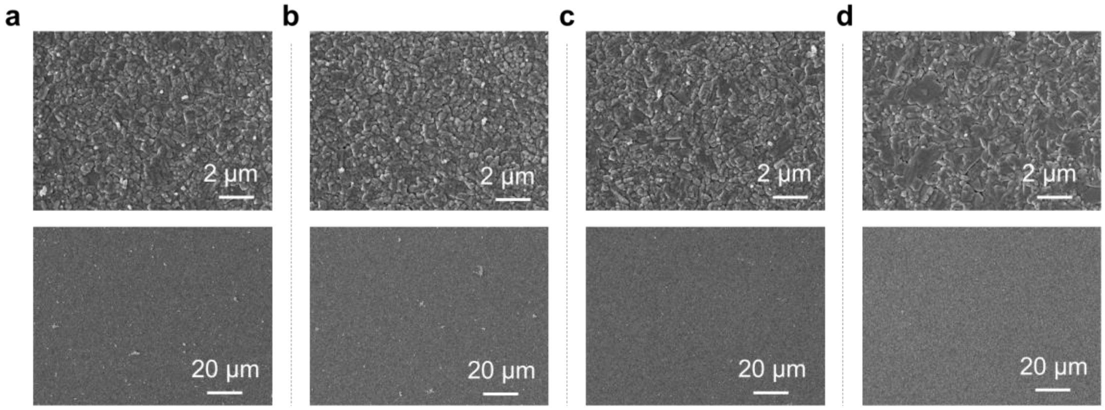


**Supplementary Fig. 6 |** Effect of rocking rate of the CdS solution bath. **a-d**, SEM images of CdS surfaces prepared under different rocking rates: 45 times/min (**a**), 30 times/min (**b**), 15 times/min (**c**), and no rocking (**d**). The images revealed that the CdS particles originated from the rocking motion of CdS solution, which stirred the particles formed in the solution and caused them to adhere to the CIGS surface.

Supplementary Fig. 5 demonstrated that no large particles were formed on the AZO/glass surface and AZO/CIGS surface. However, quite a number of large particles could be found on the AZO/CdS/CIGS surface whenever there were particles on the CdS surface to start with. As seen in Supplementary Fig. 6, depending on the motion of the CdS precursor solution, the number of large size particles on the CdS surface could be controlled. To eliminate these large particles, the rocking rate of CdS precursor solution had to be greatly reduced. On CdS surfaces without large particles, the CIGS device finished with AZO layer was also particle-free.

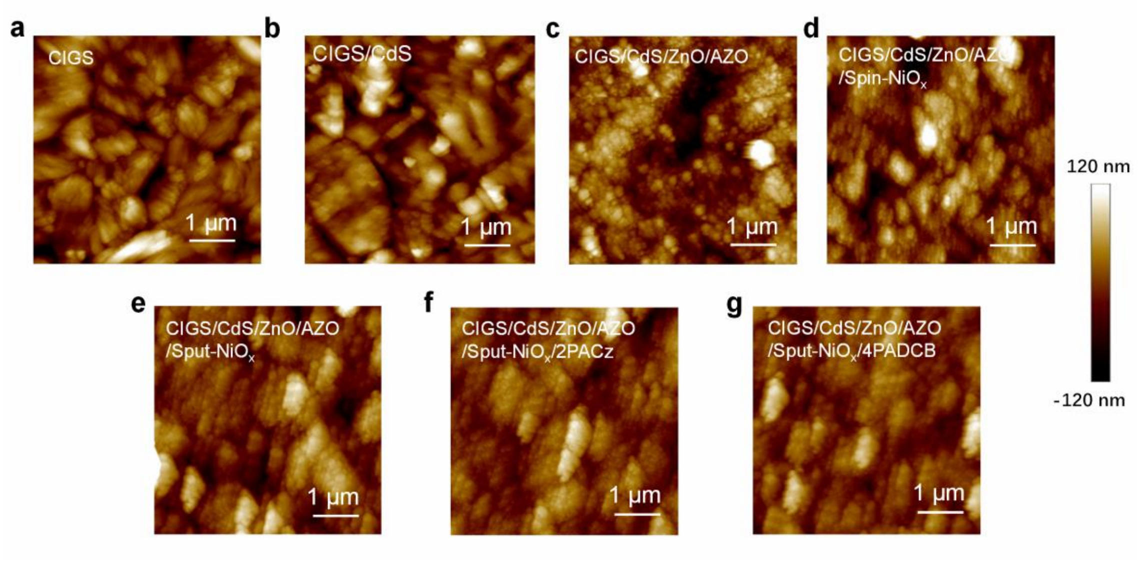


**Supplementary Fig. 7** | Surface morphology images of various layers by atomic force microscopy. CIGS (**a**), CIGS/CdS (**b**),CIGS/CdS/ZnO/AZO (**c**), CIGS/CdS/ZnO/AZO/Spin-$NiO_x$ (**d**), CIGS/CdS/ZnO/AZO/Sput-$NiO_x$ (**e**), CIGS/CdS/ZnO/AZO/Sput-$NiO_x$/2PACz (**f**), CIGS/CdS/ZnO/AZO/Sput-$NiO_x$/4PADCB (**g**). Quantitative roughness values for these layers are summarized in Supplementary Table 2.

Shown in Supplementary Fig. 7, the active CIGS layer co-evaporated onto a flat Mo-coated glass substrate exhibited a root-mean-square ($R$q) surface roughness of ~33.6 nm. With care taken on the rocking rate of the CdS precursor solution, the CIGS device ending with CdS and AZO layer had a slightly increased roughness to ~36.8 nm and ~38.9 nm. The sputtered $NiO_x$ layer smoothed the surface a bit to ~33.9 nm roughness. With 2PACz or 4PADCB SAMs deposited on the $NiO_x$ layer, the surface roughness was further reduced to ~28.5 nm and 26.5 nm, possibly due to stacked molecules in the pits. This magnitude of surface roughness was tolerable for fabricating decent perovskite/CIGS tandems, as demonstrated previously[2], and no further mechanical and chemical polishing was required[3]. In summary, eliminating large CdS particles ensures uniform coverage, suppresses shunting, and promotes conformal layer growth, thereby enhancing device performance and fabrication reliability.

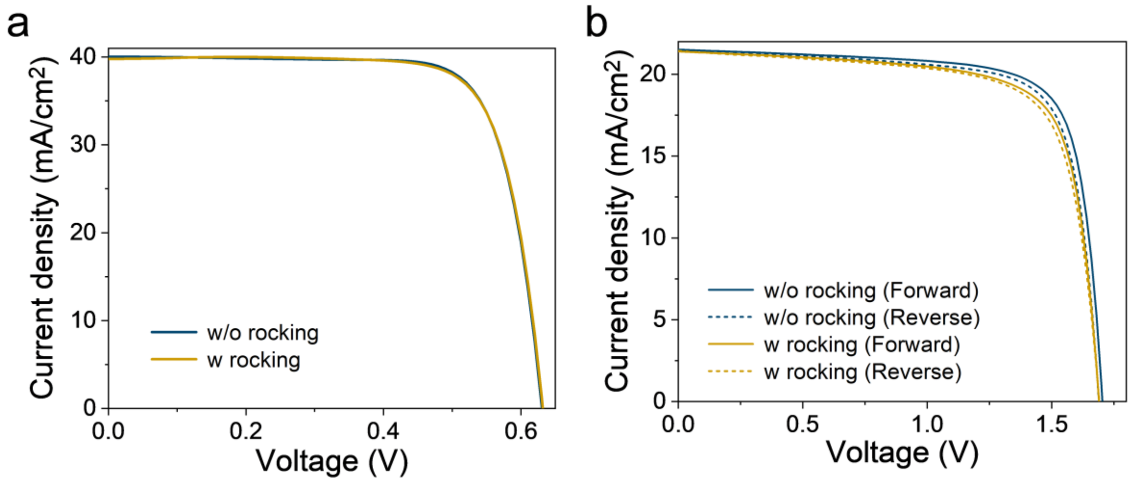


**Supplementary Fig. 8 |** *J*-*V* curves of CIGS cell (**a**) and tandem cell (**b**) with different CdS deposition modes.

Supplementary Fig. 6 shows that static CdS deposition (without rocking) results in a smoother surface with lower roughness. This improved morphology has negligible impact on the CIGS subcells (Supplementary Fig. 8a). However, reduction of shunt pathways and improved interface quality enhance performance, yielding higher open-circuit voltage ($V_{OC}$) and fill factor (FF) for the tandem devices. Comparison of *J*-*V* characteristics under different CdS deposition conditions (Supplementary Fig. 8b) confirms that improved film quality translates to better tandem device performance

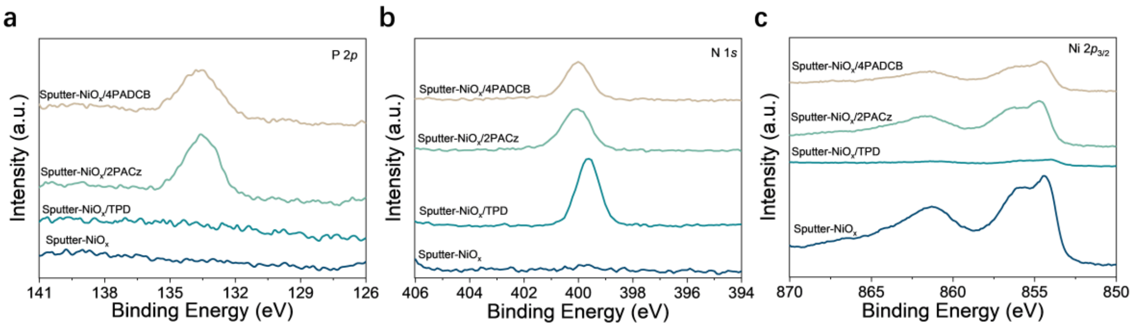


**Supplementary Fig. 9 |** The XPS spectra of P 2*p* (**a**), N 1*s* (**b**), and Ni 2*p* (**c**) of Sput, Sput/TPD, Sput/2PACz and Sput/4PADCB films. The Ni signals for Sput/TPD sample are barely visible because of the screening by thick TPD layer.

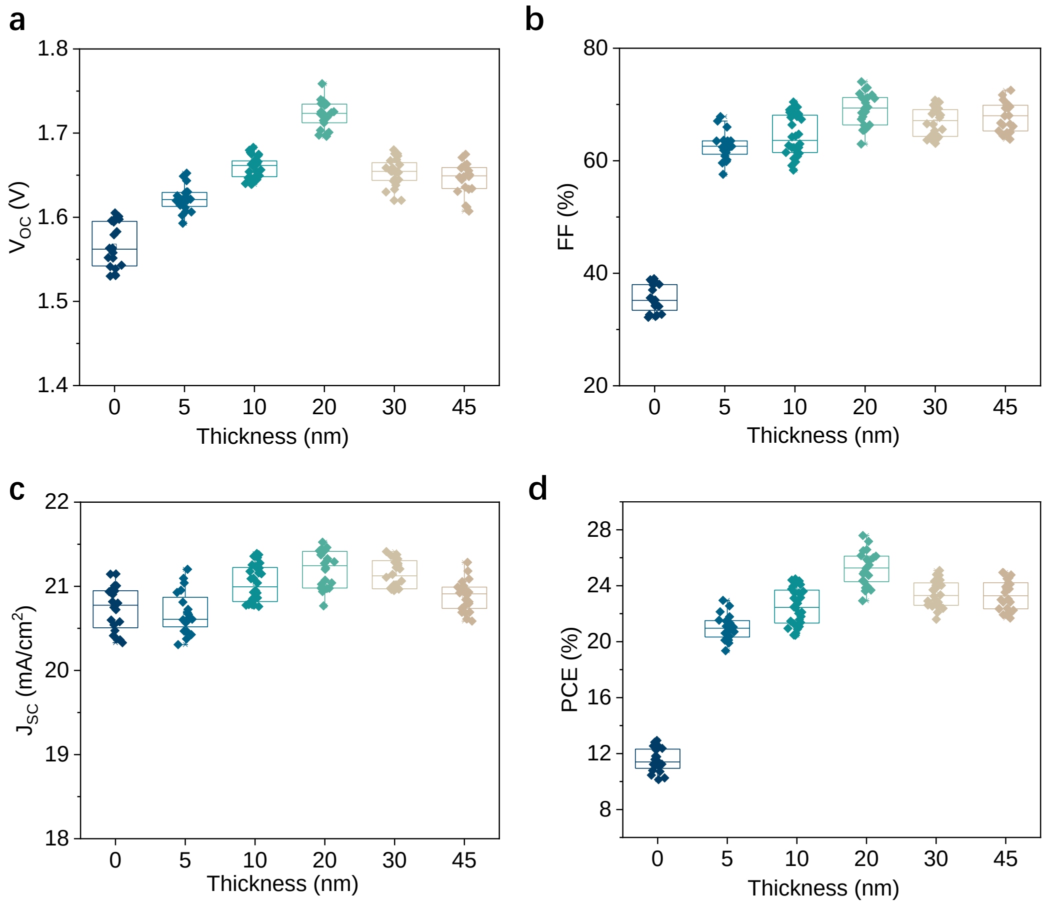


**Supplementary Fig. 10** | Optimization of sputtered $NiO_x$ layer thickness. $V_{OC}$ (**a**), FF (**b**), $J_{SC}$ (**c**), and PCE (**d**) as functions of $NiO_x$ thickness. All tandem devices incorporated 4PADCB in the hybrid HTLs. Each data point corresponds to a statistical set ($n \geq 20$). The open circle, top and bottom whiskers, and box indicate the mean, minimum and maximum values, and the 25%-75% interquartile range, respectively.

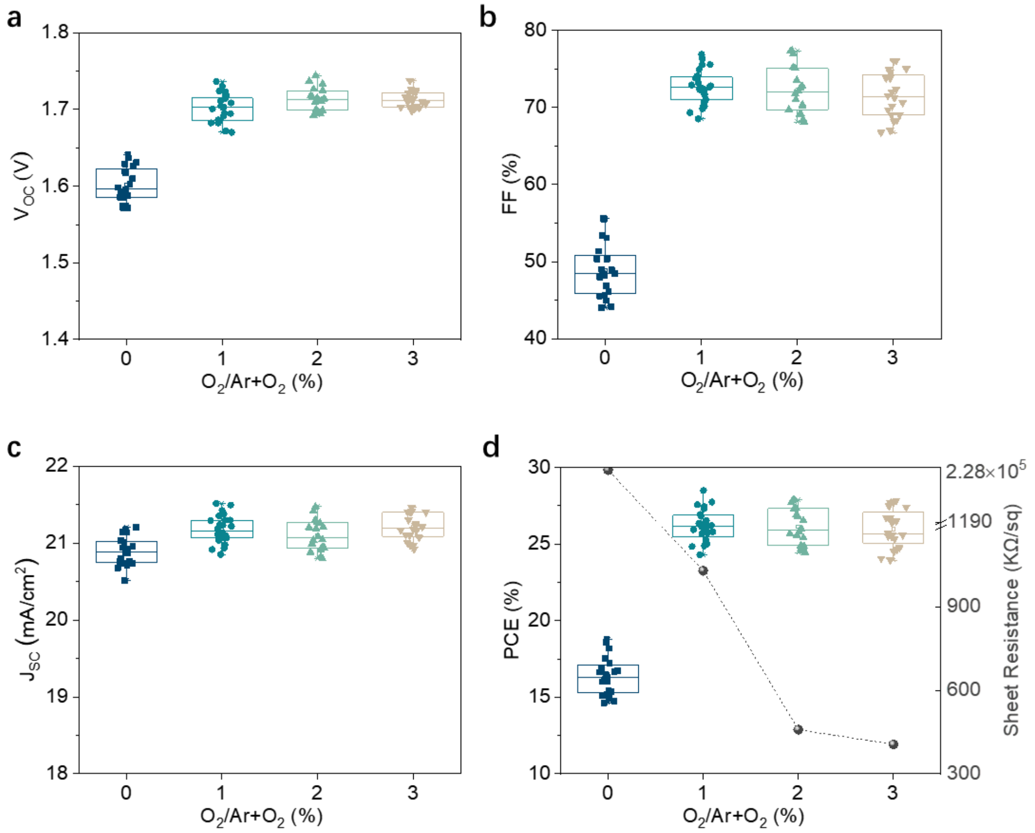


**Supplementary Fig. 11** | Optimization of oxygen content in the sputtering atmosphere for $NiO_x$. $V_{OC}$ (**a**), FF (**b**), $J_{SC}$ (**c**), and PCE (**d**) as functions of $O_2/(Ar+O_2)$ rate during sputtering. The corresponding sheet resistance is also shown in (d). The layer thickness is set at 20 nm. Each data point corresponds to a statistical set (n ≥ 18). The open circle, top and bottom whiskers, and box indicate the mean, minimum and maximum values, and the 25%-75% interquartile range, respectively.

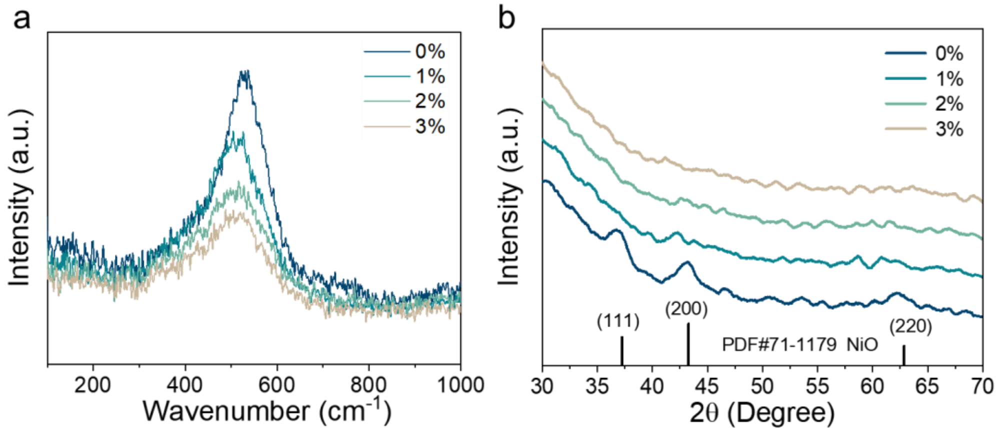


**Supplementary Fig. 12** | Raman spectra (**a**) and GI-XRD (**b**) of $NiO_x$ under different $O_{2}$/Ar+$O_2$ conditions.

Supplementary Fig. 12 shows the Raman and XRD results for different oxygen contents. From the Raman spectra, we can see a clear nickel oxide characteristic peak near 530 $cm^{-1}$. As the oxygen content increases, the Raman characteristic peak shifts to the left and the intensity decreases [4,5]. From the XRD results, the films prepared under different conditions all had a $NiO_x$ peak near 43° (although with decreasing intensity with oxygen content), but no Ni peak at 44.5°, indicating that we obtained a pure nickel oxide film without metallic nickel[6]. To be noticed, in this study, the $NiO_x$ layers were deposited using a $NiO_x$ ceramic target instead of a metallic Ni target, enabling better control over film stoichiometry and reproducibility.

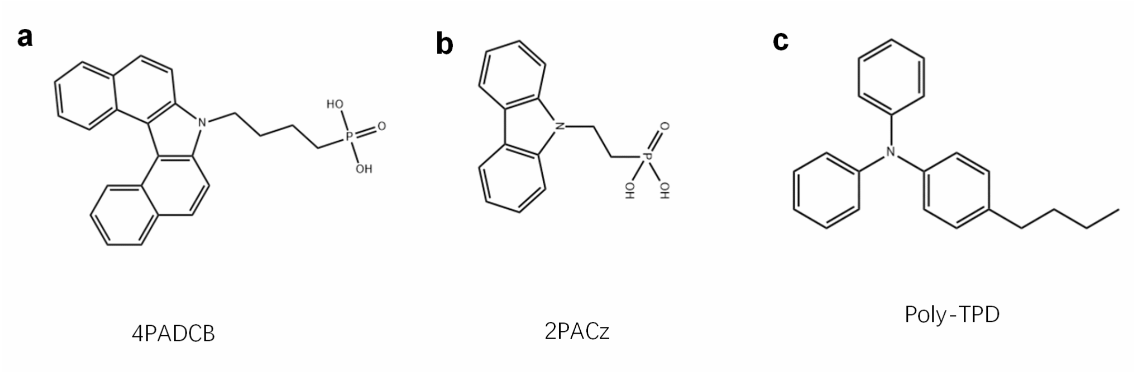


**Supplementary Fig. 13 |** Structure of three different hole transport materials: 4PADCB (**a**), 2PACz (**b**), and Poly-TPD (**c**).

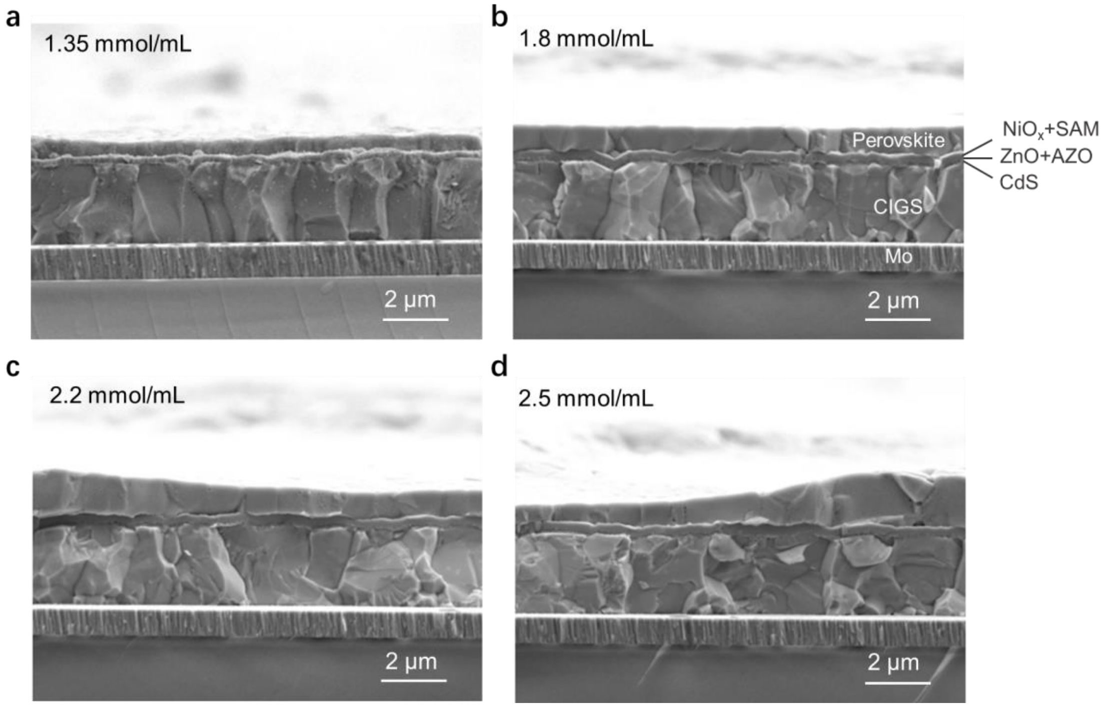


**Supplementary Fig. 14 |** Effect of perovskite precursor solution concentration: 1.35 mmol/mL (**a**), 1.8 mmol/mL (**b**), 2.2 mmol/mL (**c**), and 2.5 mmol/mL (**d**). Cross-sectional SEM images for perovskite/CIGS tandem solar cells with four different precursor concentrations, as labeled in the panels. The perovskite layers appear at the top of each image. As the solution concentration increases, the average perovskite layer thickness also increases. However, concentrations exceeding 1.8 mmol/mL result in significant non-uniformity. The optimal perovskite thickness is approximately 500 nm (at 1.35 mmol/mL) for single-junction perovskite solar cells, and 900 nm (at 1.8 mmol/mL) for the perovskite top subcells in tandem devices.

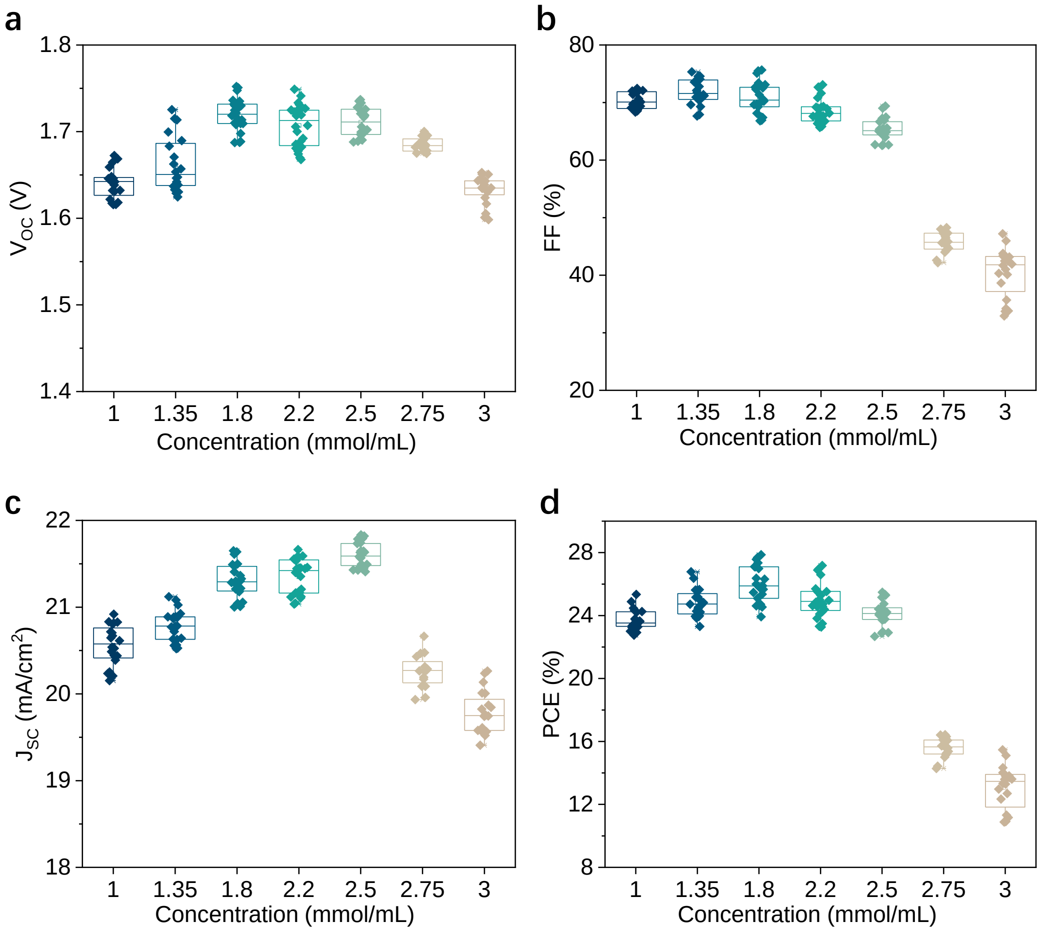


**Supplementary Fig. 15** | Optimization of the perovskite precursor solution concentration for perovskite/CIGS tandem solar cells. $V_{OC}$ (**a**), FF (**b**), $J_{SC}$ (**c**), and PCE (**d**) as functions of perovskite precursor solution concentration. The optimal solution concentration was identified as 1.8 mmol/mL. Each data point corresponds to a statistical set ($n \geq 16$). The open circle, top and bottom whiskers, and box indicate the mean, minimum and maximum values, and the 25%-75% interquartile range, respectively.

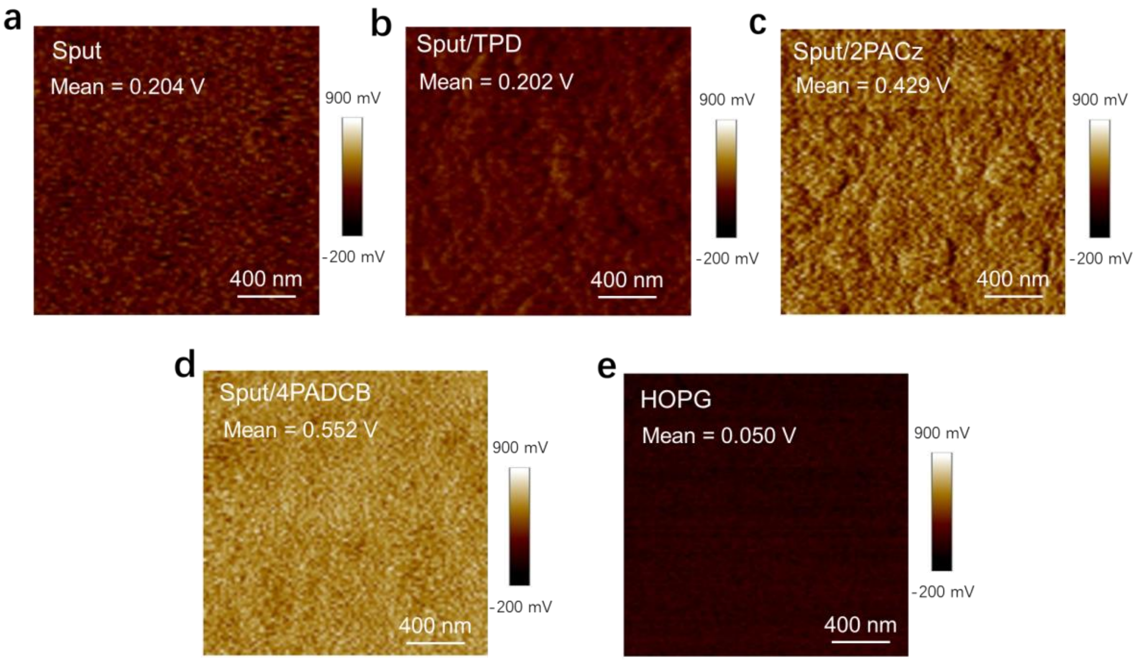


**Supplementary Fig. 16 |** Surface potential images measured by Kelvin probe force microscopy in air. **a-e**, Surface potential images for sputtered $NiO_x$ (**a**), TPD on sputtered $NiO_x$ (**b**), 2PACz on sputtered $NiO_x$ (**c**), 4PADCB on sputtered $NiO_x$ (**d**), highly oriented pyrolytic graphite (HOPG) (**e**).

All films except HOPG were directly deposited on the AZO surface of CIGS solar cells. Corresponding AFM images are shown in Supplementary Fig. 7. The average contact potential difference (CPD) over the imaged area were respectively: 204 mV for sputtered $NiO_x$, 202 mV for TPD on sputtered $NiO_x$, 429 mV for 2PACz on sputtered $NiO_x$, and 552 mV for 4PADCB on sputtered $NiO_x$. Using a reference work function of 4.500 eV for HOPG, the surface work functions of the samples were determined to be 4.654 eV, 4.652 eV, 4.879 eV, and 5.002 eV for sputtered $NiO_x$, Sput/TPD, Sput/2PACz, and Sput/4PACDB respectively. These results align with work function values obtained from ultraviolet photoelectron spectroscopy (UPS) in Supplementary Fig. 20.

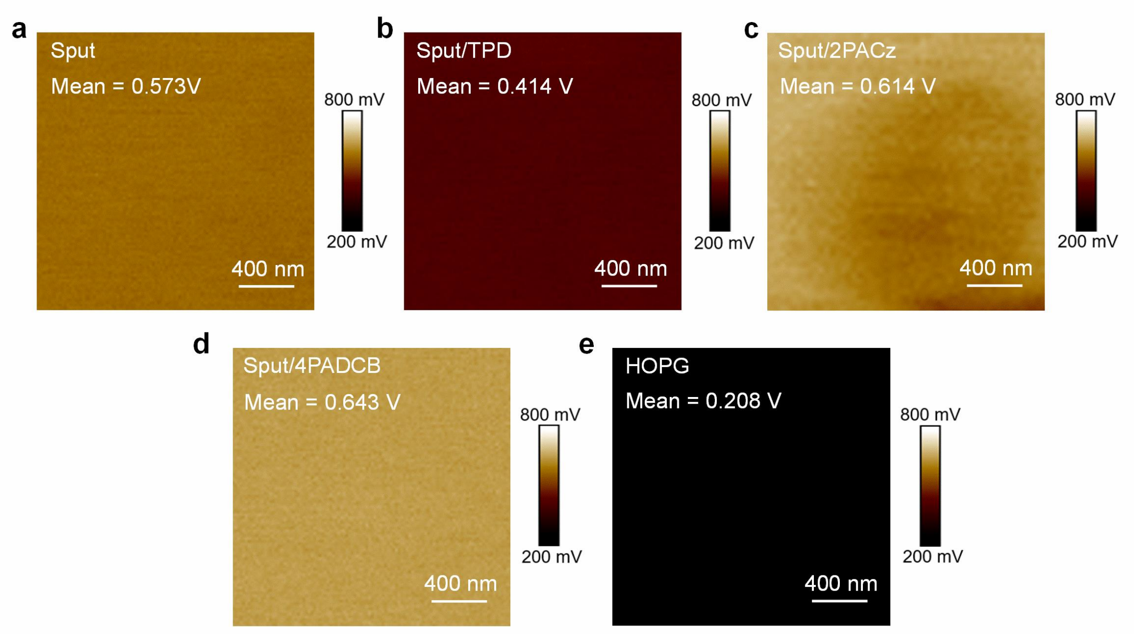


**Supplementary Fig. 17** | Surface potential images measured by Kelvin probe force microscopy under $N_2$ atmosphere. **a**-**e**, Surface potential images for sputtered $NiO_x$ (**a**), TPD on sputtered $NiO_x$ (**b**), 2PACz on sputtered $NiO_x$ (**c**), 4PADCB on sputtered $NiO_x$ (**d**), highly oriented pyrolytic graphite (HOPG) (**e**).

The CPD values of $NiO_x$, $NiO_x$/TPD, $NiO_x$/2PACz, $NiO_x$/4PADCB, and HOPG under $N_2$ atmosphere were 0.573, 0.414, 0.614, 0.643, and 0.208 V, respectively. Using a reference work function of 4.500 eV for HOPG, the surface work functions of the samples were determined to be 4.865 eV, 4.706 eV, 4.906 eV, and 4.935 eV for sputtered $NiO_x$, Sput/TPD, Sput/2PACz, and Sput/4PACDB respectively. These results reveal the CPD measurement can be affected by environment conditions, but the work function values obtained remain little dependent on the environment and are in line with results from ultraviolet photoelectron spectroscopy (UPS) in Supplementary Fig. 20.

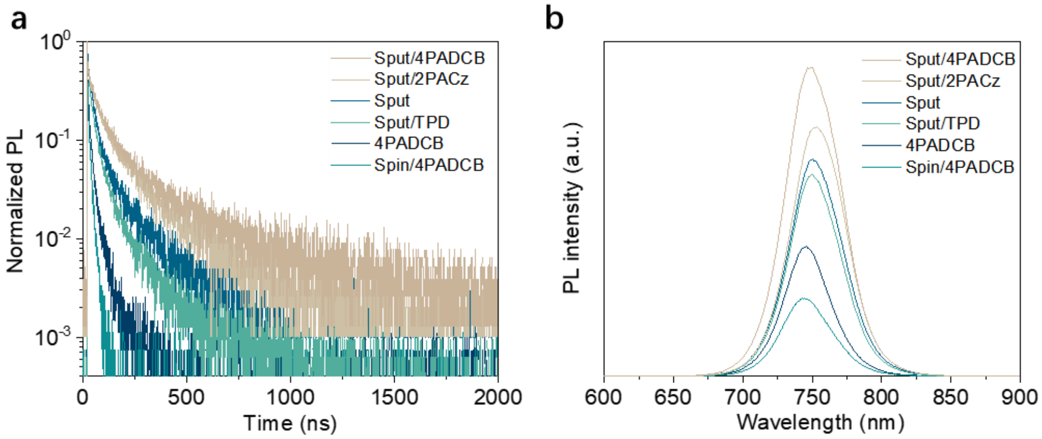


**Supplementary Fig. 18 |** Transient and steady-state photoluminescence spectroscopy over large areas. TRPL spectra of perovskite films on different HTLs (**a**), Steady-state PL spectra of perovskite films on different HTLs (**b**). Carrier lifetimes were obtained by bi-exponential fitting of the TRPL decay curves, with the results summarized in Supplementary Table 5.

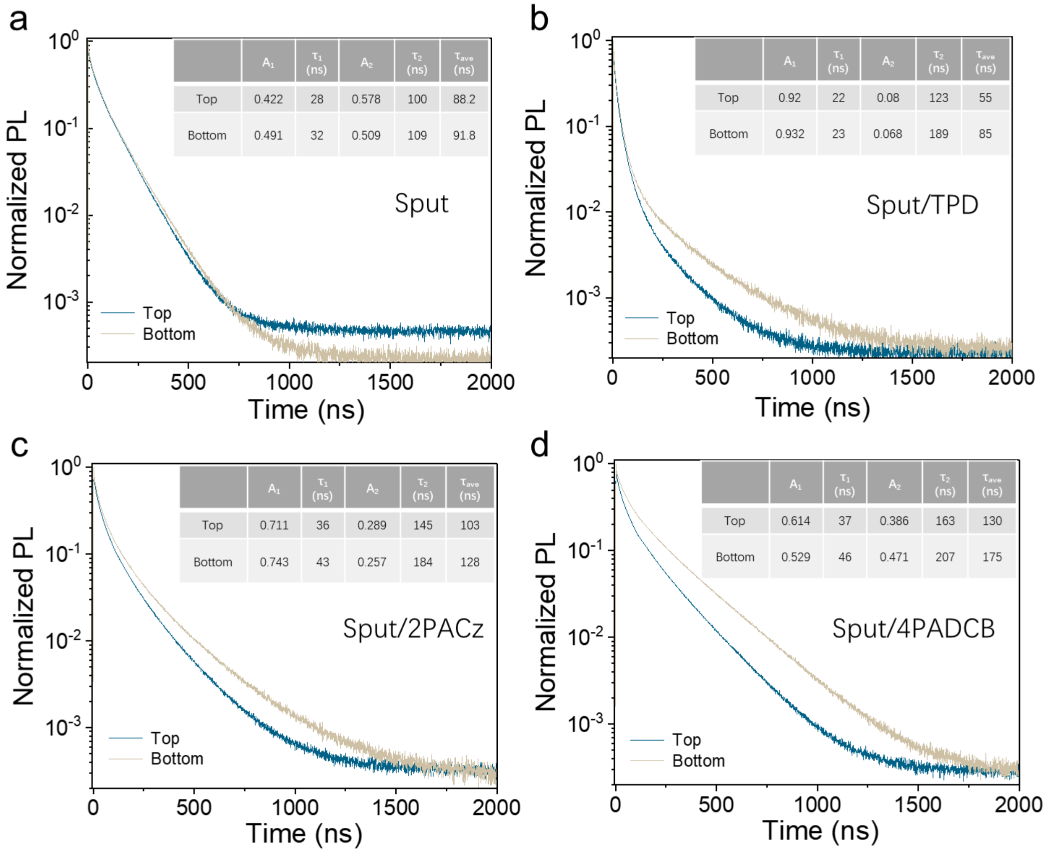


**Supplementary Fig. 19** | TRPL attenuation of front and back illuminated samples with different structures. Sput (**a**), Sput/TPD (**b**), Sput/2PACz (**c**), and Sput/4PADCB (**d**).

Supplementary Fig. 19 shows TRPL measurements under illumination from both the glass side and the perovskite side. While slight differences were observed between the two configurations[7], both exhibited consistent decay trends, reflecting similar carrier dynamics.

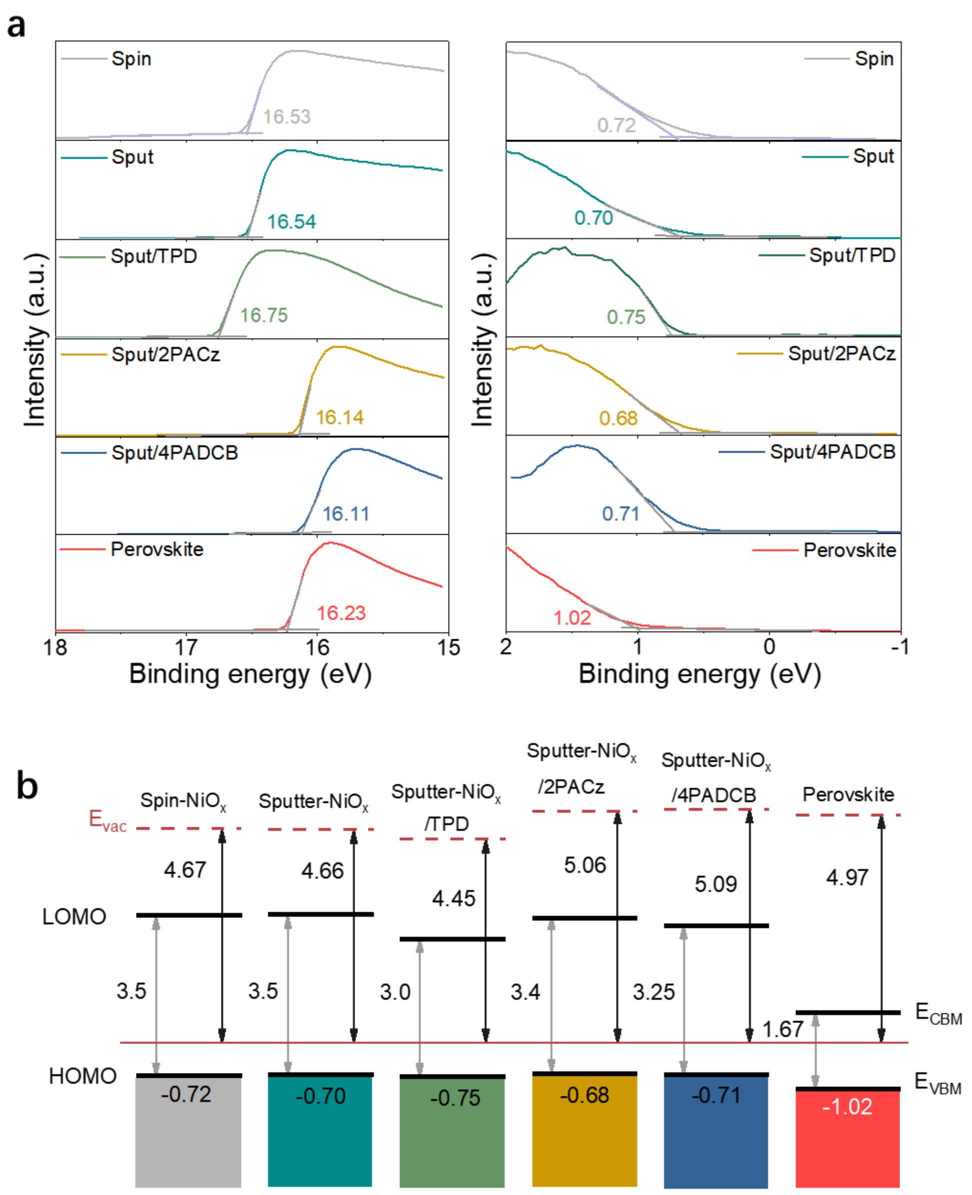


**Supplementary Fig. 20 |** UPS spectra and energy level alignment of various HTLs and the perovskite layer. UPS spectra for six different samples. The secondary electron cut-off energy and the VMB/HOMO position with respect to Fermi level could be extracted directly by extrapolations (**a**), Energy level diagram illustrating the relative energy level positions for various HTLs and the perovskite layer. The LUMO/CBM positions were obtained using the optical energy gap. The light source used was He-I with a photon energy of 21.20 eV (**b**).

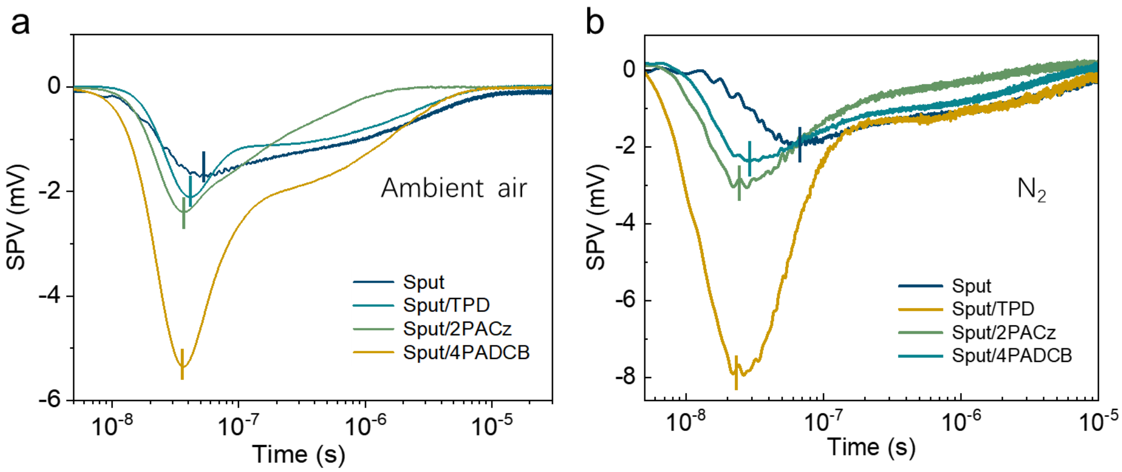


**Supplementary Fig. 21** | Hole transport dynamics detected by transient surface photovoltage (tr-SPV) for ITO/HTL/perovskite/Au-Electrode half-stacks under ambient air (**a**) and $N_2$ atmosphere (**b**). The HTLs used in the half-stacks were Sput, Sput/TPD, Sput/2PACz, and Sput/4PADCB. The deduced characteristic times are listed in Supplementary Table 6 and 7.

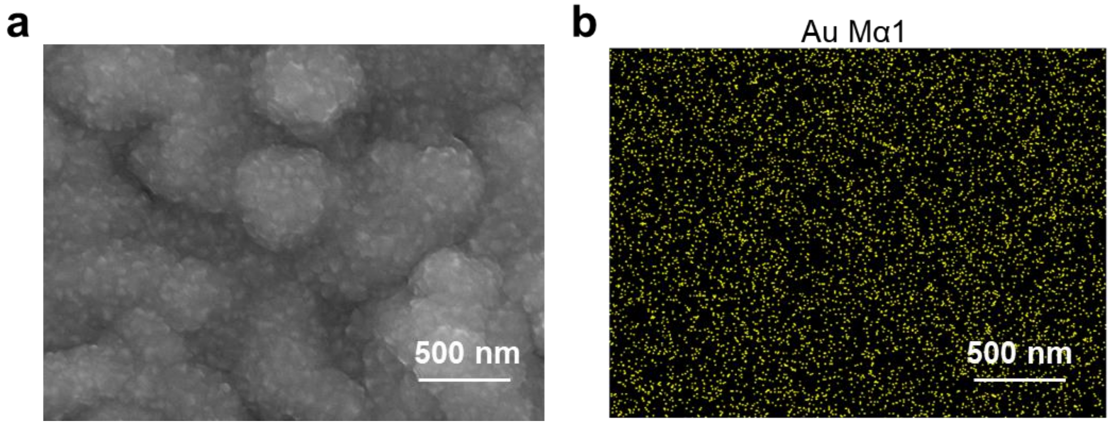


**Supplementary Fig. 22 |** Characterization of Au particles in the ultrathin film. **a-b,** SEM image (**a**) and EDX (energy dispersive X-ray spectroscopy) image of a 0.6 nm-thick Au film on AZO surface (**b**), showing that the Au film is discontinuous and consists of discrete particles.

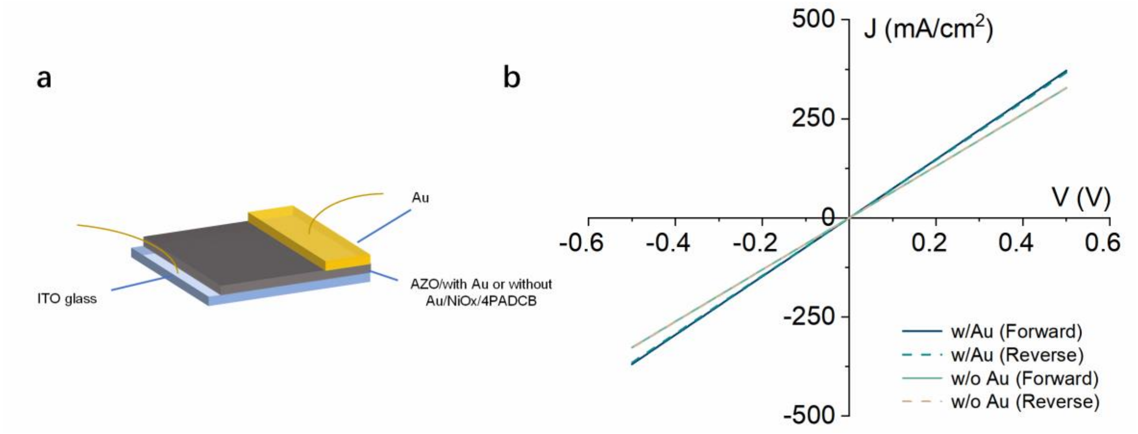


**Supplementary Fig. 23 |** Schematic diagram of *J-V* measurement of AZO/$NiO_x$/4PADCB and AZO/Au (0.6 nm) /$NiO_x$/4PADCB junctions (**a**), and *J-V* characteristics of AZO/$NiO_x$/4PADCB and AZO/Au (0.6 nm) /$NiO_x$/4PADCB junctions (**b**).

The ITO electrode is located on the AZO side, while the Au electrode is on the $NiO_x$ or $NiO_x$/4PADCB side. The "w/o Au (Forward)" and "w/o Au (Reverse)" curves overlap well, indicating negligible hysteresis. The higher current density observed in the presence of Au particles suggests that Au introduces an additional recombination channel.

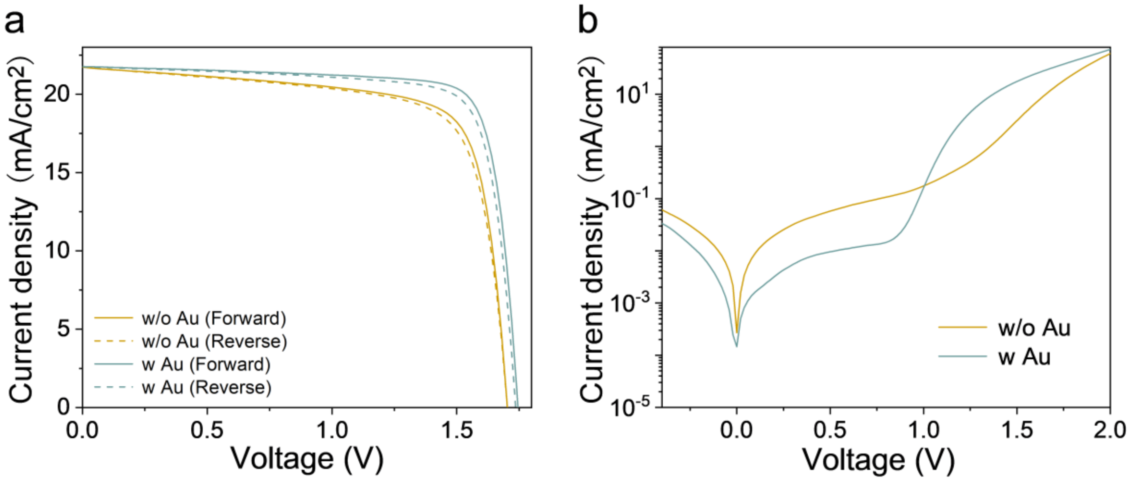


**Supplementary Fig. 24** | *J*-*V* curves (**a**), and dark *J*-*V* curves (**b**) of tandem devices with and without gold layers.

Supplementary Fig. 24 presents the *J*-*V* and dark *J*-*V* curves of tandem devices with and without the Au layer. The addition of Au results in notable increases in both $V_{OC}$ and FF, attributed to reduced series resistance and enhanced shunt resistance. The increased recombination of electrons and holes in the IRL eliminates the accumulation of un-recombined charge carriers at the junction, thereby reducing the reverse built-in electric field. The reduced charge carrier accumulation within the IRL due to the incorporation of the Au layer leads to more efficient extraction of holes from the perovskite/4PADCB/$NiO_x$ interface, thus reducing the residence time of holes and consequently the number of minority carrier recombination. A similar argument applies to electrons at the CIGS/CdS interface. As a result, higher FF and $V_{OC}$ values are achieved in the tandem devices.

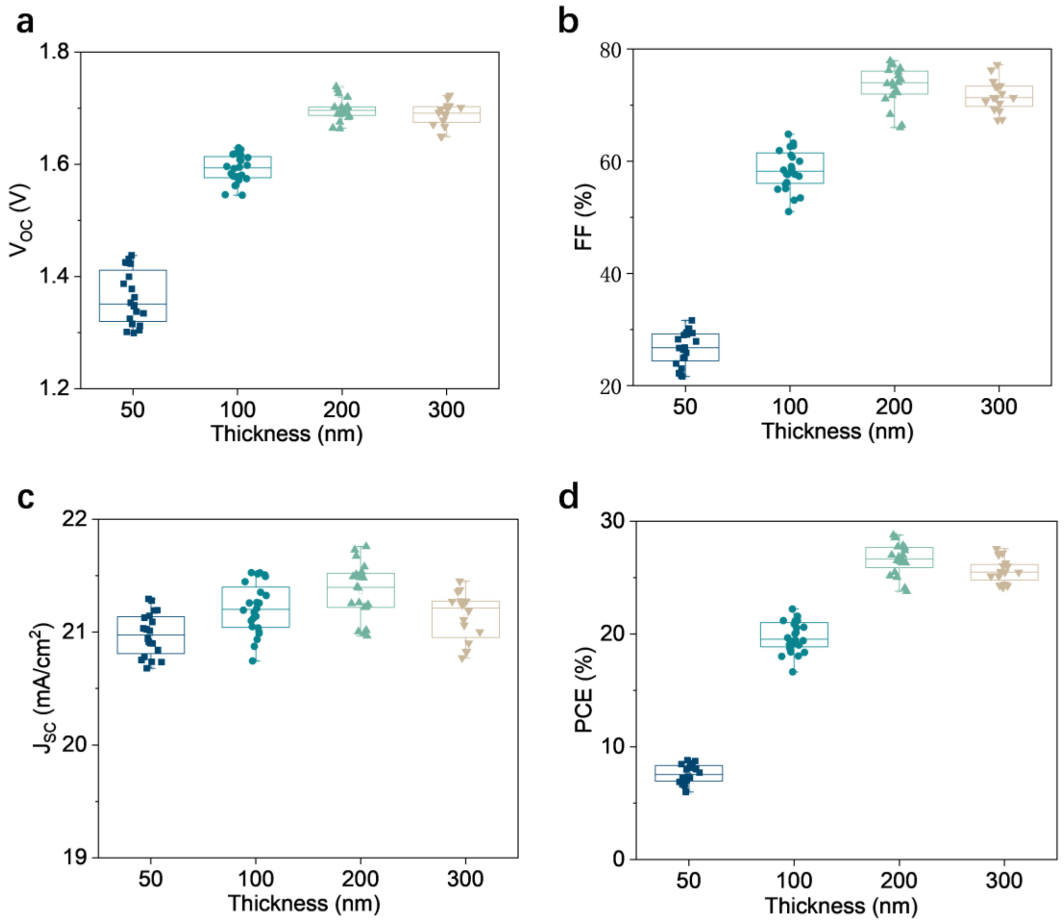


**Supplementary Fig. 25** | Optimization of AZO layer thickness. $V_{OC}$ (**a**), FF (**b**), $J_{SC}$ (**c**), PCE (**d**) as functions of the AZO thickness. The tandem devices achieved optimal performance with an AZO thickness of 200 nm. Each data point corresponds to a statistical set ($n \geq 16$). The open circle, top and bottom whiskers, and box indicate the mean, minimum and maximum values, and the 25%-75% interquartile range, respectively.

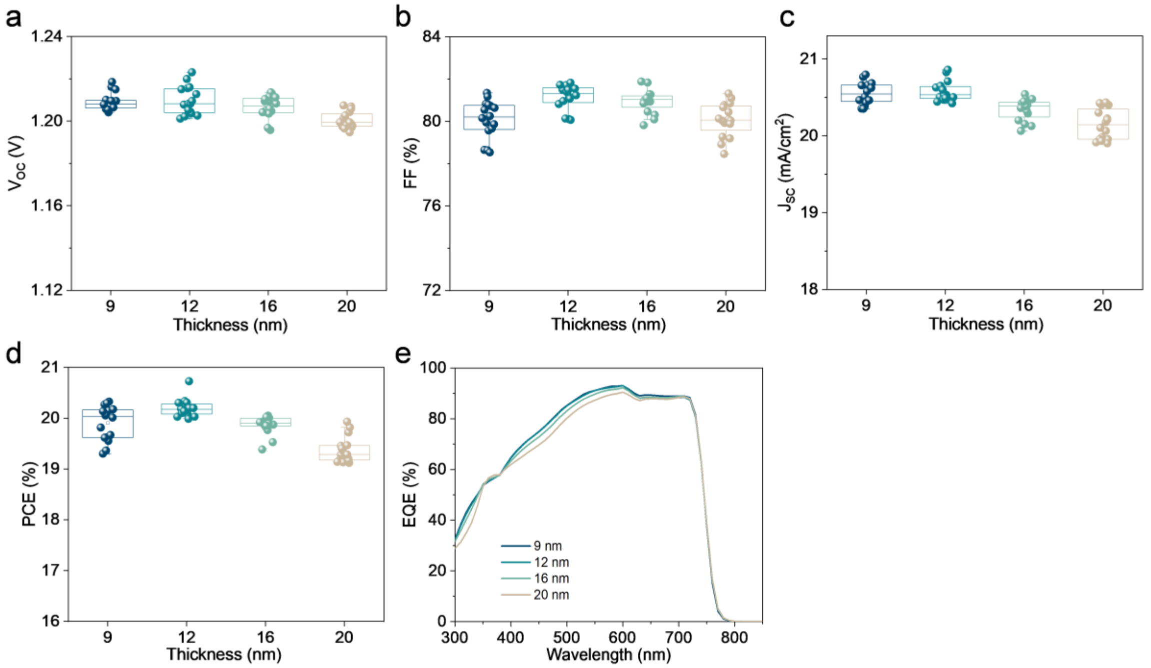


**Supplementary Fig. 26** | Effect of $C_{60}$ thickness on single-junction semi-transparent perovskite performance. $V_{OC}$ (**a**), FF (**b**), $J_{SC}$ (**c**), PCE (**d**). Each data point corresponds to a statistical set (n ≥ 16). (e) Effect of $C_{60}$ thickness on the EQE spectrum of semitransparent perovskite solar cells. The open circle, top and bottom whiskers, and box indicate the mean, minimum and maximum values, and the 25%-75% interquartile range, respectively.

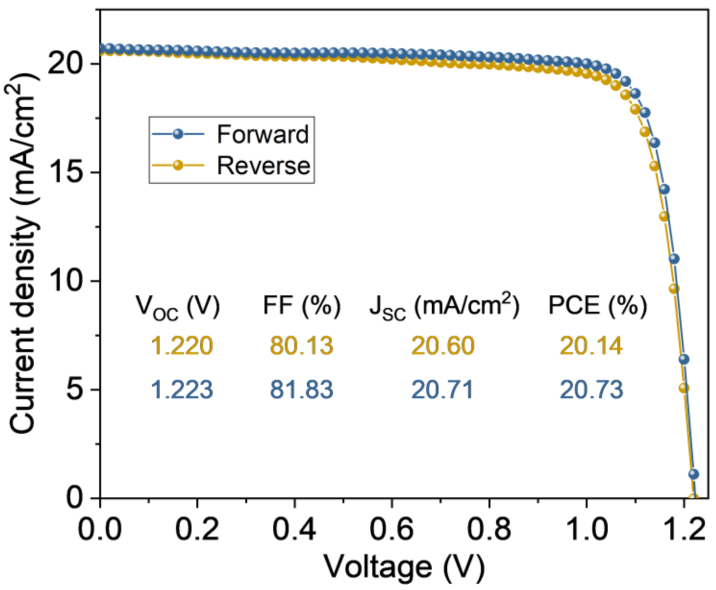


**Supplementary Fig. 27 |** Semi-transparent perovskite *J*-*V* curves with top illumination.

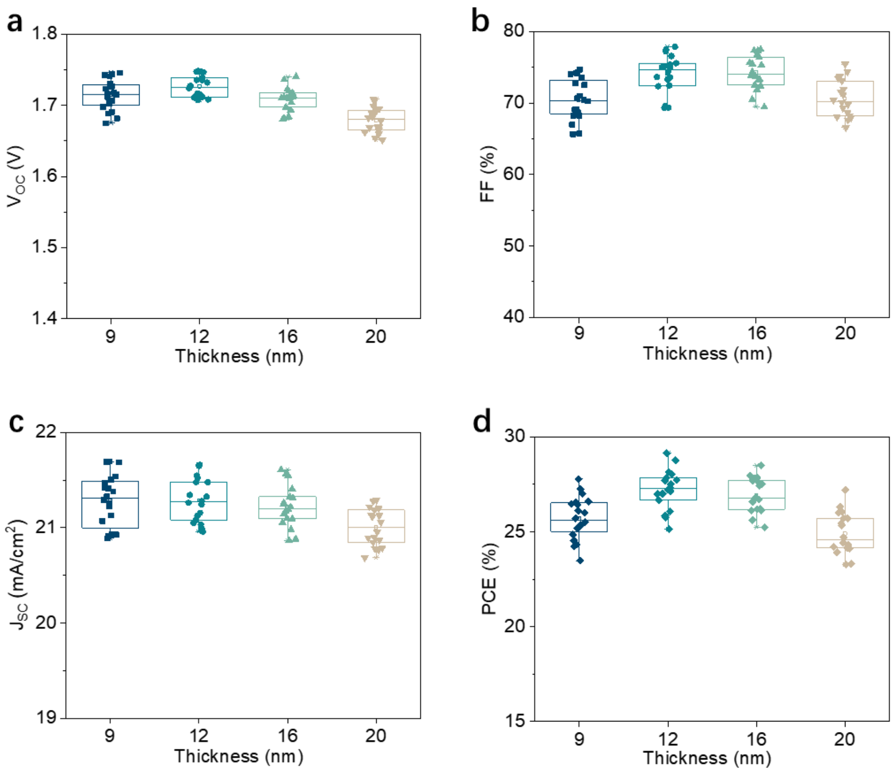


**Supplementary Fig. 28 |** Effect of $C_{60}$ thickness on tandem device performance. Impact of $C_{60}$ thickness on key performance parameters of tandem devices: (**a**) $V_{OC}$, (**b**) FF, (**c**) $J_{SC}$, and (**d**) PCE. Each data point corresponds to a statistical set ($n \geq 18$). The open circle, top and bottom whiskers, and box indicate the mean, minimum and maximum values, and the 25%-75% interquartile range, respectively.

In the current tandem structure, incident light must pass through the $C_{60}$ layer (part of the ETL) before reaching the perovskite layer. A thicker $C_{60}$ layer causes parasitic absorption in the ultraviolet region, reducing the photocurrent of the perovskite top subcell. Conversely, a thinner $C_{60}$ layer results in decreased $V_{OC}$ and FF. Therefore, an optimal thickness of approximately 12 nm, thinner than the ~20 nm typically used in stand-alone perovskite solar cells, was selected.

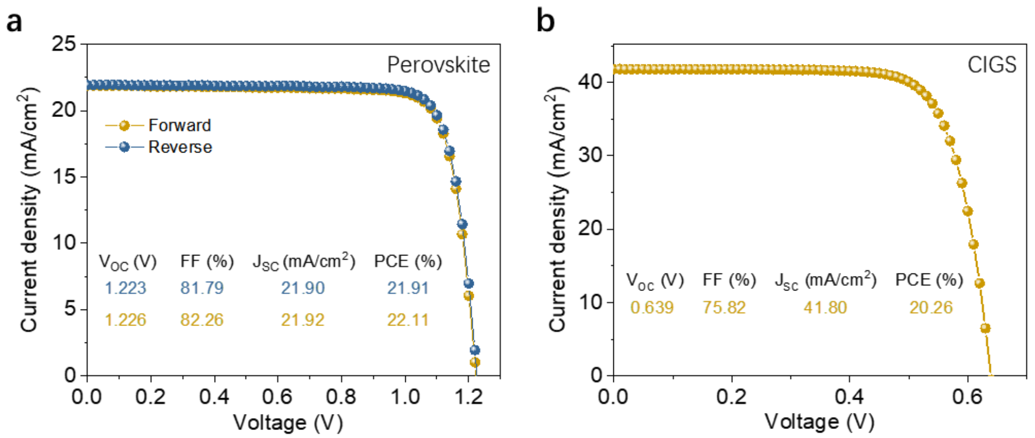


**Supplementary Fig. 29** | *J*-*V* characteristics of the top-performing stand-alone perovskite top cell (**a**) and CIGS bottom cell (**b**)**.** The perovskite device shown includes an opaque metallic electrode.

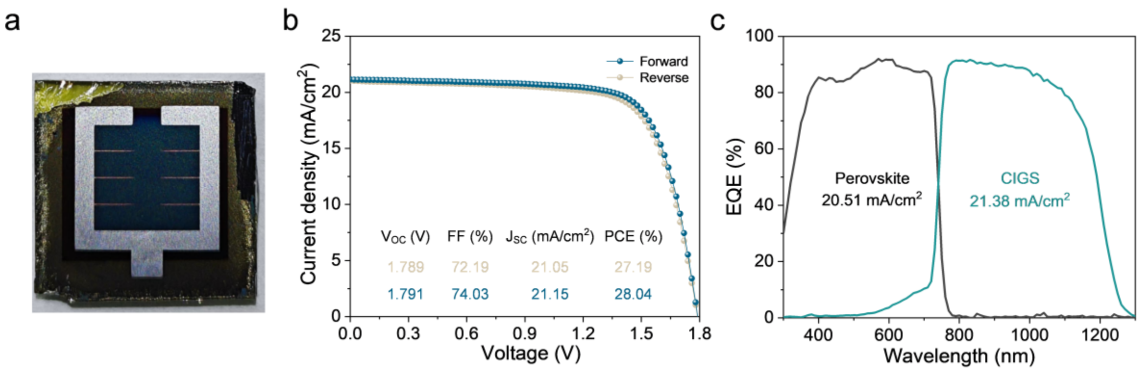


**Supplementary Fig. 30 |** Photograph of the perovskite/CIGS tandem solar cell (**a**), *J*-*V* curves (**b**), and EQE curves (**c**) of the tandem device with an area of 0.51 $cm^2$.

As shown in Supplementary Fig. 30, a large-area (0.51 $cm^2$) perovskite/CIGS tandem solar cell was prepared (a), achieving an efficiency of ~28% (b), with consistent EQE spectra observed across the device (c).

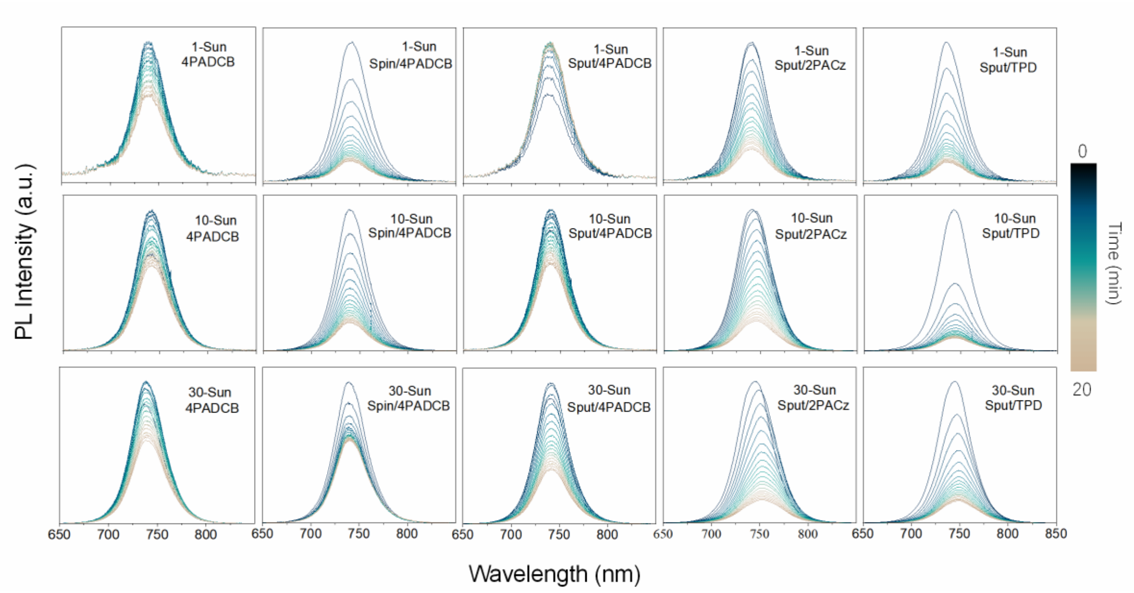


**Supplementary Fig. 31 |** Evolution of relative intensity of PL spectra for perovskite films on different HTLs (4PADCB, Spin/4PADCB, Sput/TPD, Sput/2PACz, and Sput/4PADCB) under equivalent 1-sun, 10-sun, and 30-sun illumination over 20 minutes.

Under one sun illumination, only the Sput/4PADCB combination exhibits an increase in PL intensity with minimal change over time, indicating enhanced photostability. This is consistent with reports in the literature[8]. However, when the illumination intensity is increased to 10 and 30 suns, the PL intensity for all hole transport layer combinations decreases or shifts over time. While certain data points show fluctuations, likely due to the multifaceted factors affecting device stability, the overall trend consistently indicates that 4PADCB demonstrates comparatively better stability than 2PACz and TPD, as supported by both PL intensity and spectral evolution.

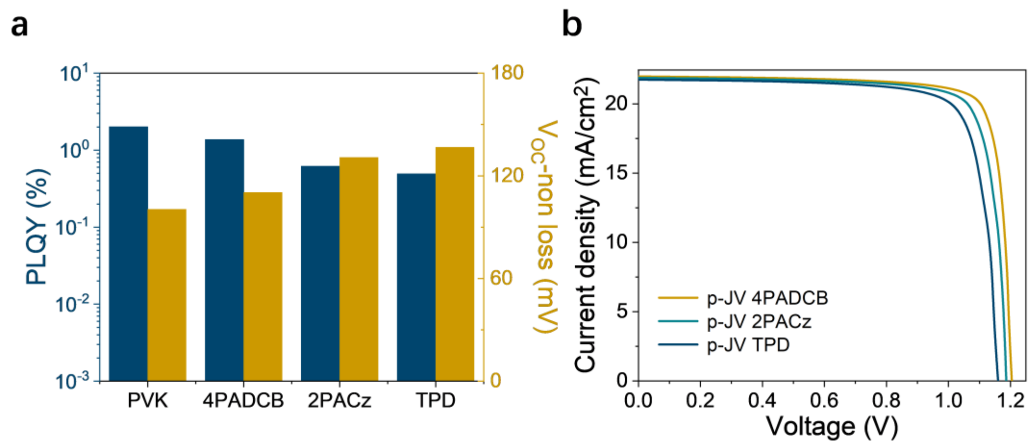


**Supplementary Fig. 32 |** PLQY values of perovskite film deposited on glass and different hole transport materials and the corresponding $V_{OC}$ non-radiative recombination loss calculated from the PLQY value (**a**), and pseudo *J-V* curves (**b**).

We prepared perovskite films on glass and three different hole transport materials and measured the PLQY of the samples using a 365 nm laser in an integrating sphere. The PLQY results of perovskite films without HTL (PVK) and on 4PADCB, 2PACz, and TPD were 2.01%, 1.37%, 0.62%, and 0.49%, respectively. The relationship between PLQY and QFLS in perovskite thin films can be described by the following equation:

$$\mathrm{QFLS} = \mathrm{QFLS}_{\mathrm{rad}} + k_B T \ln(\mathrm{PLQY}) \quad (1)$$

The non-radiative recombination loss in the $V_{OC}$ can be calculated by the following equation:

$$\Delta \mathrm{V}_{\mathrm{OC}} = \frac{(\mathrm{QFLS}_{\mathrm{rad}} - \mathrm{QFLS})}{q} = -\frac{k_B T \ln(\mathrm{PLQY})}{q} \quad (2)$$

In the above equation, $\mathrm{QFLS}_{\mathrm{rad}}$ represents the QFLS for the perovskite layer when only radiative recombination occurs, $k_B$ is the Boltzmann constant, and $T$ is the temperature.

We calculated the $V_{OC}$ losses of without HTL, with 4PADCB, 2PACz, and TPD based on the above equation to be 100.5, 110.3, 130.7, and 136.8 mV, respectively. This indicates that in comparison to other HTLs, 4PADCB can reduce non-radiative recombination, improve carrier transport efficiency, and thus have smaller $V_{OC}$ losses. Consistently, the pseudo *J-V* analysis (Supplementary Fig. 32b) confirms that employing 4PADCB reduces transport losses and suppresses non-radiative recombination, thereby improving overall device performance.

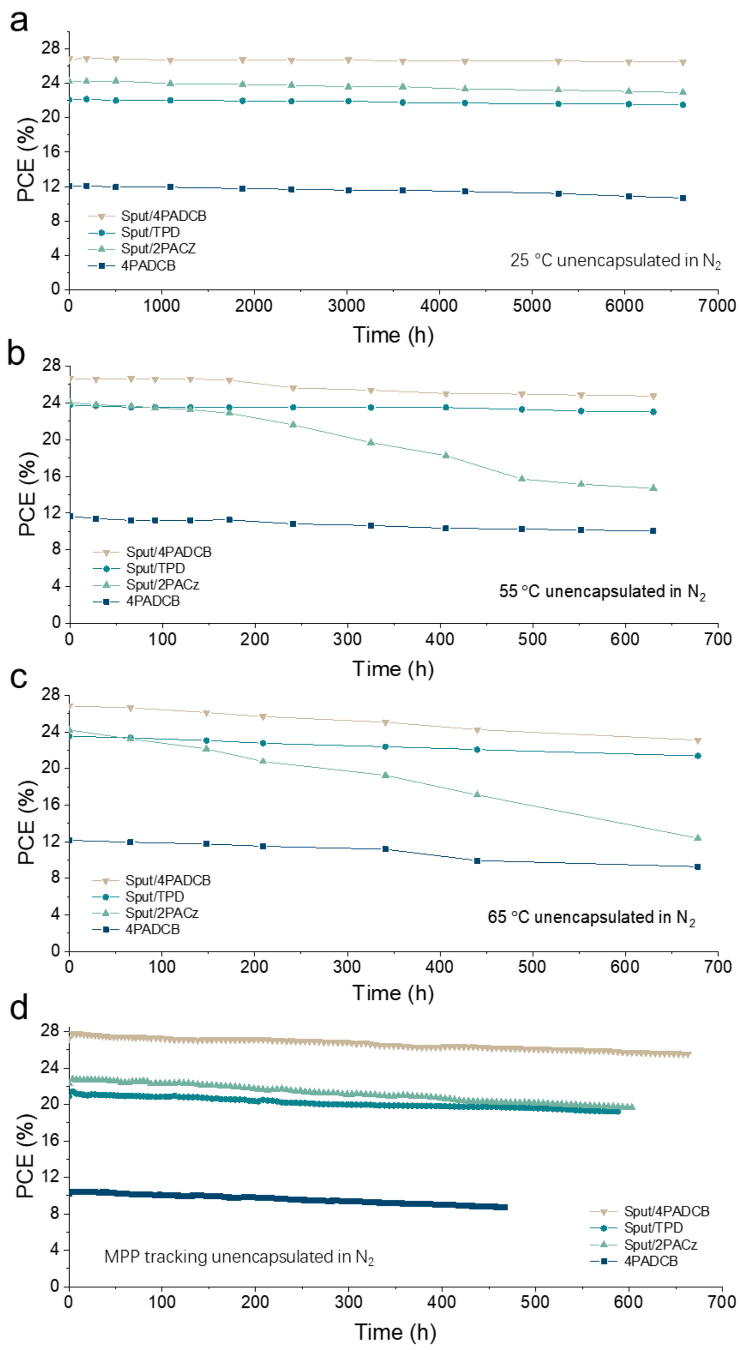


**Supplementary Fig. 33 |** Stability testing of absolute efficiency. Long-term storage stability of monolithic perovskite/CIGS tandem devices at room temperature (25°C) in an $N_2$ atmosphere (**a**), Thermal stability of tandem devices at 55°C in an $N_2$ atmosphere (**b**), Thermal stability of tandem devices at 65°C in an $N_2$ atmosphere (**c**), Continuous MPPT test for unencapsulated tandem devices (**d**).

**Supplementary Table 1.** Comparison of subcell bandgaps, IRL structures, and performance parameters among the previous monolithic perovskite/CIGS tandem solar cells with record-efficiencies.

| Top (eV) | Bottom (eV) | IRL | $V_{OC}$ (V) | $J_{SC}$ (mA/cm$^2$) | FF (%) | PCE (%) | Ref |
|---|---|---|---|---|---|---|---|
| 1.72 | 1.04 | ITO/PEDOT:PSS | 1.45 | 12.7 | 56.6 | 10.9 | [9] |
| 1.59 | 1.00 | AZO(160nm) /PEDOT:PSS | 1.346 | 12.9 | 63.5 | 11.03 | [10] |
| 1.59 | 1.08 | BZO/ITO(300 nm) /PTAA | 1.774 | 17.3 | 73.1 | 22.43* | [3] |
| 1.64 | 1.09 | AZO/$NiO_x$(10 nm) /PTAA | 1.58 | 18.0 | 76.0 | 21.6 | [11] |
| 1.60 | 1.09 | AZO(140 nm) /MeO-2PACz | 1.68 | 19.17 | 71.9 | 23.16 (23.26 ±0.75) * | [12] |
| 1.68 | 1.10 | AZO(60 nm) /Me-4PACz | 1.77 | 18.8 | 71.2 | 23.70 (24.2)* | [13] |
| 1.59 | 1.03 | IZO(20 nm) /$NiO_x$(15 nm) /2PACz | 1.59 (1.57) | 19.4 (21.1) | 75.5 (75.2) | 23.5* (24.9) | [2] |
| 1.67 | 1.01 | AZO(200 nm) /Au(0.6 nm) /$NiO_x$(20 nm) /4PADCB | 1.731 (1.745) | 21.67 (21.76) | 80.2 (80.9) | 30.1* (30.71) | This work |

Note: * indicates measurements performed by accredited independent institutions.

**Supplementary Table 2.** Root mean square surface roughness (*R*q) for perovskite/CIGS tandems processed at different stages. The values are calculated from Supplementary Fig. 7. Both spin-coating and sputtering deposition of a layer of $NiO_x$ can reduce surface roughness to some extent. Application of SAMs on sputtered $NiO_x$ can further reduce the surface roughness. The smoothened surface is beneficial for high-quality perovskite subcell construction.

| Device processed at different stage | *R*q (nm) |
|---|---|
| CIGS | 33.6 |
| CIGS/CdS | 36.8 |
| CIGS/CdS/ZnO/AZO | 38.9 |
| CIGS/CdS/ZnO/AZO/Spin-$NiO_x$ | 37.8 |
| CIGS/CdS/ZnO/AZO/Sput-$NiO_x$ | 33.9 |
| CIGS/CdS/ZnO/AZO/Sput-$NiO_x$/2PACz | 28.5 |
| CIGS/CdS/ZnO/AZO/Sput-$NiO_x$/4PADCB | 26.5 |

**Supplementary Table 3**. Summary of the electrical properties of $NiO_x$ films prepared by sputtering.

| $O_2$/Ar+$O_2$ (%) | Sheet Resistance (Ω/sq) | Resistivity (Ω·cm) | Carrier concentration ($cm^{-3}$) | Conductivity (S/cm) |
|---|---|---|---|---|
| 0 | $2.28*10^{8}$ | $1.12*10^{3}$ | $3.12*10^{15}$ | $8.93*10^{-4}$ |
| 1 | $1.03*10^{6}$ | 5.15 | $2.05*10^{17}$ | $1.95*10^{-1}$ |
| 2 | $4.59*10^{5}$ | 2.29 | $4.48*10^{18}$ | $4.37*10^{-1}$ |
| 3 | $4.06*10^{5}$ | 2.03 | $7.48*10^{18}$ | $4.92*10^{-1}$ |

**Supplementary Table 4.** Statistical analysis of surface potential values from KPFM measurements on $NiO_x$/4PADCB samples under $N_2$ atmosphere.

| **Sample No.** | **Voltage (V)** | **Sample No.** | **Voltage (V)** |
|---|---|---|---|
| 1 | 0.677 | 9 | 0.666 |
| 2 | 0.657 | 10 | 0.667 |
| 3 | 0.669 | 11 | 0.680 |
| 4 | 0.673 | 12 | 0.661 |
| 5 | 0.650 | 13 | 0.671 |
| 6 | 0.674 | 14 | 0.678 |
| 7 | 0.665 | 15 | 0.680 |
| 8 | 0.668 | 16 | 0.683 |
| **Average** | | 0.670 | |
| **Standard Deviation** | | 0.009 | |

**Supplementary Table 5.** Carrier lifetime measured by TRPL decay curves. The fitting parameters with a bi-exponential decay equation are listed below.

| HTL | $A_1$ | $\tau_1$ (ns) | $A_2$ | $\tau_2$ (ns) | $\tau_{ave}$ (ns) |
|---|---|---|---|---|---|
| 4PADCB | 0.864 | 15.0 | 0.136 | 58.0 | 31 |
| Sput | 0.882 | 30 | 0.118 | 195 | 107 |
| Spin/4PADCB | 0.799 | 9.8 | 0.201 | 24.0 | 15 |
| Sput/TPD | 0.893 | 28.0 | 0.107 | 146 | 74 |
| Sput/2PACz | 0.812 | 58 | 0.188 | 260 | 161 |
| Sput/4PADCB | 0.811 | 60 | 0.189 | 337 | 217 |

**Supplementary Table 6.** Carrier extraction time measured by tr-SPV curves, corresponding to Supplementary Fig. 21a. The carrier extraction time is defined as the duration required to reach the maximum surface photovoltage (SPV). The decay time is determined by fitting the data to a bi-exponential decay equation, as described in the Methods section.

| Sample | $\tau_{extraction}$ (ns) | $\tau_1$ (ns) | $\tau_2$ (μs) |
|---|---|---|---|
| Sput | 53.3 | 107.6 | 3.08 |
| Sput/TPD | 41.4 | 22.0 | 2.63 |
| Sput/2PACz | 36.8 | 63.9 | 0.53 |
| Sput/4PADCB | 36.0 | 33.8 | 1.86 |

**Supplementary Table 7**. Carrier extraction time measured by tr-SPV curves under $N_2$ atmosphere, corresponding to Supplementary Fig. 21b. The carrier extraction time is defined as the duration required to reach the maximum surface photovoltage (SPV). The decay time is determined by fitting the data to a bi-exponential decay equation, as described in the Methods section.

| Sample | $\tau_{extraction}$ (ns) | $\tau_1$ (ns) | $\tau_2$ (μs) |
|---|---|---|---|
| Sput | 67.3 | 120.5 | 5.80 |
| Sput/TPD | 29.2 | 66.4 | 3.53 |
| Sput/2PACz | 24.5 | 63.8 | 1.66 |
| Sput/4PADCB | 23.3 | 36.8 | 6.41 |

**Supplementary Table 8.** Performance survey of the four-terminal perovskite/CIGS tandems.

| Filtered CIGS | | | | | PSC | | 4-T | |
|---|---|---|---|---|---|---|---|---|
| $E_g$ (eV) | $V_{OC}$ (V) | $J_{SC}$ (mA/cm$^2$) | FF (%) | PCE (%) | $E_g$ (eV) | PCE (%) | PCE (%) | Year |
| 1.14 | 0.560 | 10.2 | 69.6 | 4.0 | 1.55 | 11.5 | 15.5 | 2015[14] |
| 1.10 | 0.682 | 10.9 | 78.8 | 5.9 | 1.58 | 12.7 | 18.6 | 2015[15] |
| 1.15 | 0.661 | 14.4 | 77.4 | 7.4 | 1.57 | 12.1 | 19.5 | 2015[16] |
| 1.15 | 0.669 | 12.1 | 73.6 | 6.0 | 1.55 | 16.1 | 22.1 | 2016[17] |
| 1.00 | 0.428 | 15.3 | 73.1 | 4.8 | 1.55 | 16.1 | 20.9 | |
| 1.02 | 0.470 | 15.2 | 64.6 | 4.7 | 1.58 | 16.0 | 20.7 | 2017[18] |
| 1.16 | 0.620 | 13.0 | 72.0 | 5.8 | 1.62 | 18.1 | 23.9 | 2018[19] |
| 1.00 | 0.581 | 18.6 | 74.2 | 8.0 | 1.60 | 16.1 | 24.1 | 2019[20] |
| 1.12 | 0.715 | 15.6 | 79.2 | 8.8 | 1.68 | 17.1 | 25.9 | 2019[21] |
| 1.13 | 0.710 | 13.6 | 78.1 | 7.5 | 1.65 | 17.5 | 25.0 | 2020[22] |
| 1.00 | 0.564 | 17.5 | 73.7 | 7.3 | 1.55 | 17.3 | 24.6 | 2020[23] |
| 1.02 | 0.625 | 15.5 | 77.4 | 7.5 | 1.61 | 18.0 | 25.5 | 2022[24] |
| 1.02 | 0.623 | 13.9 | 77.4 | 6.7 | 1.55 | 19.5 | 26.2 | 2022[24] |
| 1.00 | 0.549 | 14.3 | 72.4 | 5.7 | 1.58 | 21.4 | 27.1 | 2022[25] |
| 1.13 | 0.660 | 17.7 | 78.6 | 8.8 | 1.62 | 18.5 | 27.3 | 2022[26] |
| 1.18 | 0.703 | 14.6 | 76.4 | 7.8 | 1.67 | 18.6 | 26.4 | 2023[27] |
| 1.14 | 0.674 | 15.6 | 77.1 | 8.1 | 1.67 | 18.6 | 26.7 | 2023[27] |
| 1.04 | 0.598 | 20.3 | 77.1 | 9.3 | 1.67 | 19.1 | 28.4 | 2023[27] |
| 1.00 | 0.570 | 19.3 | 73.7 | 8.1 | 1.63 | 21.8 | 29.9 | 2023[28] |
| 1.01 | 0.627 | 20.9 | 76.3 | 10.0 | 1.67 | 19.0 | 29.0 | 2024[1] |

**Supplementary Note 1.** Certification report of a monolithic perovskite/CIGS tandem solar cell. Sent to an accredited independent PV calibration laboratory, Quality Supervision & Testing Center of Chemical & Physical Power Sources of the Information Industry (QSTC), for independent measurements. The certified device had a photoactive area of 0.15 $cm^2$, which was defined via a mechanical isolation and a subtraction of the opaque electrode regions using dimension-calibrated optical microscopic image. The measurement was carried out using a calibrated class AAA solar simulator.

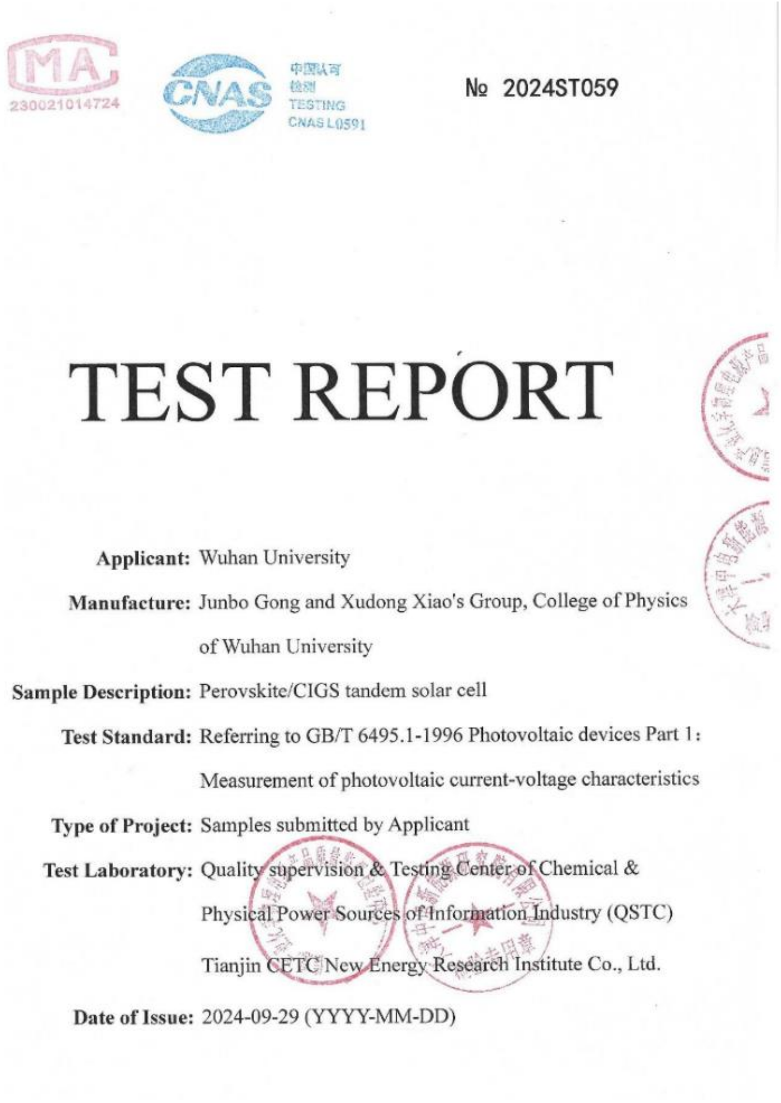

MA 230021014724 CNAS 中国认可 检测 TESTING CNAS L0591 № 2024ST059

# TEST REPORT

**Applicant:** Wuhan University

**Manufacture:** Junbo Gong and Xudong Xiao's Group, College of Physics of Wuhan University

**Sample Description:** Perovskite/CIGS tandem solar cell

**Test Standard:** Referring to GB/T 6495.1-1996 Photovoltaic devices Part 1: Measurement of photovoltaic current-voltage characteristics

**Type of Project:** Samples submitted by Applicant

**Test Laboratory:** Quality supervision & Testing Center of Chemical & Physical Power Sources of Information Industry (QSTC) Tianjin CETC New Energy Research Institute Co., Ltd.

**Date of Issue:** 2024-09-29 (YYYY-MM-DD)

№ 2024ST059

**Quality supervision & Testing Center of Chemical & Physical Power Sources of Information Industry (QSTC)**

**Tianjin CETC New Energy Research Institute Co., Ltd.**

**Announcement**

1. The legal entity of QSTC is Tianjin CETC New Energy Research Institute Co., Ltd..
2. The test report is only valid for the samples submitted by the Applicant and the related sampling batch (the sampling is made by the applicant or the relevant agencies), test data can' be used for business activities by QSTC.
3. Only the entire test report with the stamp of Tianjin CETC New Energy Research Institute Co., Ltd. is valid.
4. The test report is invalid without the signatures of authorizer, checker or drafter.
5. If there is any disagreement about the test report, please inform us in writing.
6. Tested samples will be kept for 3 months at most, if exceeding the deadline, they will be disposed according to the related regulation.
7. The position of the test report authorizer (☐the technical person in charge, ☑the quality person in charge).

☑ Lab(main site): No.6, Huake seven RD, Hi-tech Industry Development Area, Tianjin.

☐ Lab(sub site): No. 3, Lane 1, Yanshan Wei, Tianxin Community, Tangxia Town, Dongguan City, Guangdong Province.

Post Address: No.6, Huake seven RD, Haitai street, Xiqing District,

Tianjin, 300384, China.

Tel:+86-22-23959006

Fax:+86-22-23942864

**Quality supervision & Testing Center of Chemical & Physical Power Sources of Information Industry (QSTC)**

**Tianjin CETC New Energy Research Institute Co., Ltd.**

**Test Summary**

№ 2024ST059

| | |
|---|---|
| **Assignment Source:** | Entrusted by Wuhan University, one piece of Perovskite/CIGS tandem solar cell was submitted by Junbo Gong and Xudong Xiao's Group, College of Physics of Wuhan University. |
| **Test Standard:** | Referring to GB/T 6495.1-1996 Photovoltaic devices Part 1: Measurement of photovoltaic current-voltage characteristics. |
| **Test Names:** | Measurement of photovoltaic current-voltage characteristics. |
| **Test Conclusions:** | Report test data. |
| **Note:** | There is no subcontracting test;<br>More information is shown in Datasheet (see table 1～2). |

**It is blank hereinafter.**

Authorizer: [signature] Approver: [signature] Drafter: [signature]

## Quality supervision & Testing Center of Chemical & Physical Power Sources of Information Industry (QSTC)

## Tianjin CETC New Energy Research Institute Co., Ltd.

## Sample Description

**№2024ST059**

| | | | |
|---|---|---|---|
| Applicant | Wuhan University | | |
| Address | No.299, Bayi Road, Wuchang district ,Wuhan, Hubei Province | Telephone | — |
| Manufacturer | Junbo Gong and Xudong Xiao's Group, College of Physics of Wuhan University | Brand | — |
| Address | No.299, Bayi Road, Wuchang district ,Wuhan, Hubei Province | Telephone | — |
| Sample description | Perovskite/CIGS tandem solar cell | Sample type | — |
| Sample batch | — | Sample quantity | 1 |
| Sample No. | 240919-01B | | |
| Pictures | 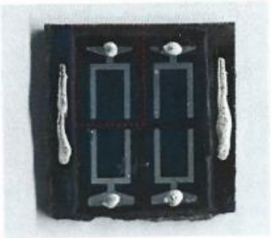<br>Pic.1: Front view (illustrated by the red boxes )<br>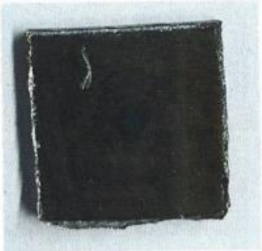<br>Pic.2: Back view | | |
| Note | | | |

Authorizer: Approver: Drafter:

**Quality supervision & Testing Center of Chemical & Physical Power Sources of Information Industry (QSTC)**

**Tianjin CETC New Energy Research Institute Co., Ltd.**

## Report Overview

| Type of project | Samples submitted by Applicant | Report No. | 2024ST059 |
|---|---|---|---|
| Sampling basis/place | —— | | |
| Test standard | Referring to GB/T 6495.1-1996 Photovoltaic devices Part 1: Measurement of photovoltaic current-voltage characteristics | | |
| Date of sample received | 2024-09-19 | Date of sample accepted | 2024-09-19 |
| Dates of tests | 2024-09-19 | | |

| No. | Test Names | Equipment Name & ID | Ambient Condition TEMP ℃ | Ambient Condition RH % | Test Verdicts | Operator |
|---|---|---|---|---|---|---|
| 1 | Measurement of photovoltaic current-voltage characteristics | Solar simulator (60410042) IR detector (34480831 WS) | 25 | 45 | Report data | He Xiwei |
| | Blank hereinafter | | | | | |
| | | | | | | |
| | | | | | | |
| | | | | | | |
| | | | | | | |
| | | | | | | |
| | | | | | | |
| | | | | | | |
| | | | | | | |
| | | | | | | |
| | | | | | | |
| | | | | | | |
| | | | | | | |
| | | | | | | |
| | | | | | | |
| | | | | | | |
| | | | | | | |
| Note | | | | | | |

Authorizer: [signature] Approver: [signature] Drafter: [signature]

**Quality supervision & Testing Center of Chemical & Physical Power Sources of Information Industry (QSTC)**

**Tianjin CETC New Energy Research Institute Co., Ltd.**

## Test results of Perovskite/CIGS tandem solar cell

Type: / №2024ST059 Table 1

| Test Item | Measurement of photovoltaic current-voltage characteristics | | | | | | |
|---|---|---|---|---|---|---|---|
| Technical requirements | Determine the current – voltage characteristics of the solar cell and an irradiance of 100 $mW \cdot cm^{-2}$, in accordance with IEC 60904-1 using a simulator class B or better conforming to the requirements of IEC 60904-9. | | | | | | |
| Sample No. | Test results | | | | | | |
| | Voc (V) | Isc (mA) | Vmp (V) | Imp (mA) | Pmax (mW) | FF (%) | η (%) |
| 240919-01B | 1.731 | 3.255 | 1.517 | 2.98 | 4.518 | 80.2 | 30.1 |
| Blank hereinafter | | | | | | | |
| | | | | | | | |
| | | | | | | | |
| | | | | | | | |
| | | | | | | | |
| | | | | | | | |
| | | | | | | | |
| | | | | | | | |
| | | | | | | | |
| | | | | | | | |
| | | | | | | | |
| | | | | | | | |
| | | | | | | | |
| | | | | | | | |
| | | | | | | | |
| | | | | | | | |
| | | | | | | | |
| | | | | | | | |
| | | | | | | | |
| Note | 1) The irradiance of solar simulator was calibrated with crystalline silicon reference cell according to Applicant's requirements.<br>2) The active area (S) of No. 240919-01B illustrated by the red boxe in Pic.1 is 0.1502 $cm^2$. The area was offered by National Institute of Metrology, China and Certificate No. is CDjc2024-08041.<br>3) $\eta = Pmax/(S \times 100\ mW \cdot cm^{-2}) \times 100\%$. | | | | | | |

Authorizer: Approver: Drafter:

**Quality supervision & Testing Center of Chemical & Physical Power Sources of Information Industry (QSTC)**

**Tianjin CETC New Energy Research Institute Co., Ltd.**

**Test results of Perovskite/CIGS tandem solar cell**

Type: /　　　　№2024ST059　Table 2

| I-V curve of No.240919-01B |
| --- |
| 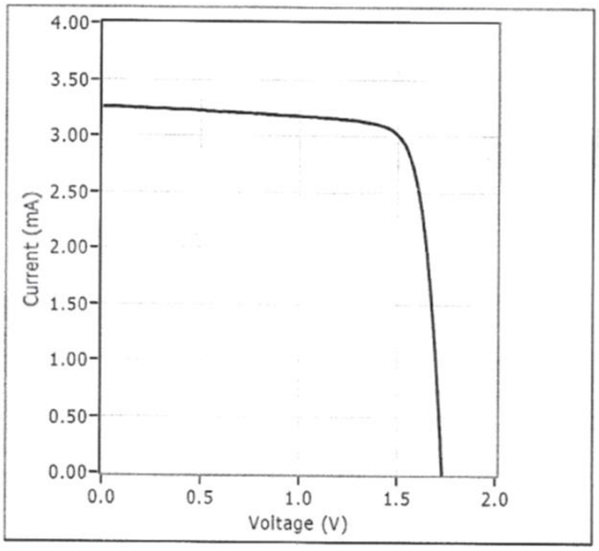 |

| Note | END OF REPORT. |
| --- | --- |

Authorizer: 　　Approver: 　　Drafter:

## Supplementary References


1. J. Zhang, Z. Ma, Y. Zhang, X. Liu, R. Li, Q. Lin, G. Fang, X. Zheng, W. Li, C. Yang, J. Li, J. Gong, X. Xiao, Highly efficient narrow bandgap Cu(In,Ga)$Se_2$ solar cells with enhanced open circuit voltage for tandem application. *Nat. Commun.* **15**, 10365 (2024).
2. M. A. Ruiz-Preciado, F. Gota, P. Fassl, I. M. Hossain, R. Singh, F. Laufer, F. Schackmar, T. Feeney, A. Farag, I. Allegro, Monolithic two-terminal perovskite/CIS tandem solar cells with efficiency approaching 25%. *ACS Energy Lett.* **7**, 2273-2281 (2022).
3. Q. Han, Y.-T. Hsieh, L. Meng, J.-L. Wu, P. Sun, E.-P. Yao, S.-Y. Chang, S.-H. Bae, T. Kato, V. Bermudez, High-performance perovskite/Cu(In,Ga)$Se_2$ monolithic tandem solar cells. *Science* **361**, 904-908 (2018).
4. M. Wang, Y. Thimont, L. Presmanes, X. Diao, A. Barnabé, The effect of the oxygen ratio control of DC reactive magnetron sputtering on as-deposited non stoichiometric NiO thin films. *Appl. Surf. Sci.* **419**, 795-801 (2017).
5. A. H. Hammad, M. S. Abdel-wahab, S. Vattamkandathil, A. R. Ansari, Influence the oxygen flow rate on the film thickness, structural, optical and photoluminescence behavior of DC sputtered NiO thin films. *Physica B: Condensed Matter* **568**, 6-12 (2019).
6. J. Richardson, X-ray diffraction study of nickel oxide reduction by hydrogen. *Applied Catalysis A: General* **246**, 137-150 (2003).
7. S.-G. Kim, J.-H. Kim, P. Ramming, Y. Zhong, K. Schötz, S. J. Kwon, S. Huettner, F. Panzer, N.-G. Park, How antisolvent miscibility affects perovskite film wrinkling and photovoltaic properties. *Nat. Commun.* **12**, 1554 (2021).
8. A. Al-Ashouri, E. Köhnen, B. Li, A. Magomedov, H. Hempel, P. Caprioglio, J. A. Márquez, A. B. Morales Vilches, E. Kasparavicius, J. A. Smith, Monolithic perovskite/silicon tandem solar cell with> 29% efficiency by enhanced hole extraction. *Science* **370**, 1300-1309 (2020).

9. T. Todorov, T. Gershon, O. Gunawan, Y. S. Lee, C. Sturdevant, L. Y. Chang, S. Guha, Monolithic Perovskite-CIGS Tandem Solar Cells via In Situ Band Gap Engineering. *Adv. Energy Mater.* **5**, 1500799 (2015).
10. Y. H. Jang, J. M. Lee, J. W. Seo, I. Kim, D.-K. Lee, Monolithic tandem solar cells comprising electrodeposited $CuInSe_2$ and perovskite solar cells with a nanoparticulate ZnO buffer layer. *J Mater Chem A* **5**, 19439-19446 (2017).
11. M. Jost, T. Bertram, D. Koushik, J. A. Marquez, M. A. Verheijen, M. D. Heinemann, E. Köhnen, A. Al-Ashouri, S. Braunger, F. Lang, B. Rech, T. Unold, M. Creatore, I. Lauermann, C. A. Kaufmann, R. Schlatmann, S. Albrecht, 21.6%-efficient monolithic perovskite/$Cu(In,Ga)Se_2$ tandem solar cells with thin conformal hole transport layers for integration on rough bottom cell surfaces. *ACS Energy Lett.* **4**, 583-590 (2019).
12. A. Al-Ashouri, A. Magomedov, M. Ross, M. Jost, M. Talaikis, G. Chistiakova, T. Bertram, J. A. Márquez, E. Köhnen, E. Kasparavicius, S. Levcenco, L. Gil-Escrig, C. J. Hages, R. Schlatmann, B. Rech, T. Malinauskas, T. Unold, C. A. Kaufmann, L. Korte, G. Niaura, V. Getautis, S. Albrecht, Conformal monolayer contacts with lossless interfaces for perovskite single junction and monolithic tandem solar cells. *Energ. Environ Sci.* **12**, 3356-3369 (2019).
13. M. Jost, E. Köhnen, A. Al-Ashouri, T. Bertram, S. Tomsic, A. Magomedov, E. Kasparavicius, T. Kodalle, B. Lipovsek, V. Getautis, R. Schlatmann, C. A. Kaufmann, S. Albrecht, M. Topic, Perovskite/CIGS tandem solar cells: from certified 24.2% toward 30% and beyond. *ACS Energy Lett.* **7**, 1298-1307 (2022).
14. Y. Yang, Q. Chen, Y.-T. Hsieh, T.-B. Song, N. D. Marco, H. Zhou, Y. J. A. n. Yang, Multilayer transparent top electrode for solution processed perovskite/$Cu(In,Ga)(Se,S)_2$ four terminal tandem solar cells. *ACS Nano* **9**, 7714-7721 (2015).
15. C. D. Bailie, M. G. Christoforo, J. P. Mailoa, A. R. Bowring, E. L. Unger, W. H. Nguyen, J. Burschka, N. Pellet, J. Z. Lee, M. Grätzel, R. Noufi, T. Buonassisi, A.

Salleo, M. D. McGehee, Semi-transparent perovskite solar cells for tandems with silicon and CIGS. *Energ. Environ Sci.* **8**, 956-963 (2015).

16. L. Kranz, A. Abate, T. Feurer, F. Fu, E. Avancini, J. Lockinger, P. Reinhard, S. M. Zakeeruddin, M. Gratzel, S. Buecheler, A. N. Tiwari, High-efficiency polycrystalline thin film tandem solar cells. *J Phys Chem Lett* **6**, 2676-2681 (2015).
17. F. Fu, T. Feurer, Thomas P. Weiss, S. Pisoni, E. Avancini, C. Andres, S. Buecheler, Ayodhya N. Tiwari, High-efficiency inverted semi-transparent planar perovskite solar cells in substrate configuration. *Nat. Energy* **2**, 1-9 (2016).
18. A. Guchhait, H. A. Dewi, S. W. Leow, H. Wang, G. Han, F. B. Suhaimi, S. Mhaisalkar, L. H. Wong, N. Mathews, Over 20% efficient CIGS-perovskite tandem solar cells. *ACS Energy Lett.* **2**, 807-812 (2017).
19. H. Shen, T. Duong, J. Peng, D. Jacobs, N. Wu, J. Gong, Y. Wu, S. K. Karuturi, X. Fu, K. Weber, X. Xiao, T. P. White, K. Catchpole, Mechanically-stacked perovskite/CIGS tandem solar cells with efficiency of 23.9% and reduced oxygen sensitivity. *Energ. Environ Sci.* **11**, 394-406 (2018).
20. T. Feurer, R. Carron, G. Torres Sevilla, F. Fu, S. Pisoni, Y. E. Romanyuk, S. Buecheler, A. N. Tiwari, Efficiency improvement of near-stoichiometric $CuInSe_2$ solar cells for application in tandem devices. *Adv. Energy Mater.* **9**, 1901428 (2019).
21. D. H. Kim, C. P. Muzzillo, J. Tong, A. F. Palmstrom, B. W. Larson, C. Choi, S. P. Harvey, S. Glynn, J. B. Whitaker, F. Zhang, Z. Li, H. Lu, M. F. A. M. van Hest, J. J. Berry, L. M. Mansfield, Y. Huang, Y. Yan, K. Zhu, Bimolecular additives improve wide-band-gap perovskites for efficient tandem solar cells with CIGS. *Joule* **3**, 1734-1745 (2019).
22. S. Gharibzadeh, I. M. Hossain, P. Fassl, B. A. Nejand, T. Abzieher, M. Schultes, E. Ahlswede, P. Jackson, M. Powalla, S. Schäfer, M. Rienäcker, T. Wietler, R. Peibst, U. Lemmer, B. S. Richards, U. W. Paetzold, 2D/3D heterostructure for semitransparent

perovskite solar cells with engineered bandgap enables efficiencies exceeding 25% in four-terminal tandems with silicon and CIGS. *Adv. Funct. Mater.* **30**, 1909919 (2020).

23. Y. Jiang, T. Feurer, R. Carron, G. T. Sevilla, T. Moser, S. Pisoni, R. Erni, M. D. Rossell, M. Ochoa, R. Hertwig, A. N. Tiwari, F. Fu, High-mobility $In_2O_3$:H electrodes for four-terminal perovskite/$CuInSe_2$ tandem solar cells. *ACS Nano* **14**, 7502-7512 (2020).
24. M. Nakamura, C. C. Lin, C. Nishiyama, K. Tada, T. Bessho, H. Segawa, Semi-transparent perovskite solar cells for four-terminal perovskite/cigs tandem solar cells. *ACS Appl. Energy Mater.* **5**, 8103-8111 (2022).
25. C. Zhang, M. Chen, F. Fu, H. Zhu, T. Feurer, W. Tian, C. Zhu, K. Zhou, S. Jin, S. M. Zakeeruddin, A. N. Tiwari, N. P. Padture, M. Grätzel, Y. Shi, CNT-based bifacial perovskite solar cells toward highly efficient 4-terminal tandem photovoltaics. *Energ. Environ Sci.* **15**, 1536-1544 (2022).
26. T. Feeney, I. M. Hossain, S. Gharibzadeh, F. Gota, R. Singh, P. Fassl, A. Mertens, A. Farag, J.-P. Becker, S. Paetel, E. Ahlswede, U. W. Paetzold, Four-terminal perovskite/copper indium gallium selenide tandem solar cells: unveiling the path to >27% in power conversion efficiency. *Sol. RRL* **6**, 2200662 (2022).
27. X. X. Liu, J. J. Zhang, L. T. Tang, J. B. Gong, W. Li, Z. Y. Ma, Z. X. Tu, Y. Y. Li, R. M. Li, X. Z. Hu, C. Shen, H. Wang, Z. P. Wang, Q. Q. Lin, G. J. Fang, S. Wang, C. Liu, Z. M. Zhang, J. M. Li, X. D. Xiao, Over 28% efficiency perovskite/$Cu(InGa)Se_2$ tandem solar cells: highly efficient sub-cells and their bandgap matching. *Energ. Environ Sci.* **16**, 5029-5042 (2023).
28. H. Liang, J. Feng, C. D. Rodríguez-Gallegos, M. Krause, X. Wang, E. Alvianto, R. Guo, H. Liu, R. K. Kothandaraman, R. Carron, A. N. Tiwari, I. M. Peters, F. Fu, Y. Hou, 29.9%-efficient, commercially viable perovskite/$CuInSe_2$ thin-film tandem solar cells. *Joule* **7**, 2859-2872 (2023).
