# Supplementary material for: Efficient perovskite/Cu(In,Ga)Se2 tandem solar cells with a composite intermediate recombination layer
Source: Nat Commun. 2025 Dec 10;17:711. doi: 10.1038/s41467-025-67350-y (PMC12820335; doi:10.1038/s41467-025-67350-y)
Supplement: Supplementary file 2 — Solar Cell Reporting Summary [file 41467_2025_67350_MOESM2_ESM.pdf]

nature portfolio

Corresponding author(s): Jianmin Li, Sheng Wang, Junbo Gong, and Xudong Xiao

Solar Cells Reporting Summary

Nature Portfolio wishes to improve the reproducibility of the work that we publish. This form is intended for publication with all accepted papers reporting the characterization of photovoltaic devices and provides structure for consistency and transparency in reporting. Some list items might not apply to an individual manuscript, but all fields must be completed for clarity.

For further information on Nature Research policies, including our data availability policy, see Authors & Referees.

Experimental design

Please check the following details are reported in the manuscript, and provide a brief description or explanation where applicable.

1. Dimensions

Area of the tested solar cells

☒ Yes — 0.15 cm2
☐ No — *Explain why this information is not reported/not relevant.*

Method used to determine the device area

☒ Yes — It has been determined by a certification laboratory
☐ No — *Explain why this information is not reported/not relevant.*

2. Current-voltage characterization

Current density-voltage (J-V) plots in both forward and backward direction

☒ Yes — Provided in this manuscript.
☐ No

Voltage scan conditions

☒ Yes — Provided in Methods section.
☐ No — *Explain why this information is not reported/not relevant.*

Test environment

☒ Yes — Provided in Methods section.
☐ No — *Explain why this information is not reported/not relevant.*

Protocol for preconditioning of the device before its characterization

☐ Yes — *Provide a description of the protocol.*
☒ No — No preconditioning was used in this work.

Stability of the J-V characteristic

☒ Yes — Provided in this manuscript.
☐ No — *Explain why this information is not reported/not relevant.*

3. Hysteresis or any other unusual behaviour

Description of the unusual behaviour observed during the characterization

☒ Yes — In the Fig. 3f.
☐ No — *Explain why this information is not reported/not relevant.*

Related experimental data

☒ Yes — In the Fig. 3f.
☐ No — *Explain why this information is not reported/not relevant.*

4. Efficiency

External quantum efficiency (EQE) or incident photons to current efficiency (IPCE)

☒ Yes — In the Fig. 3g.
☐ No — *Explain why this information is not reported/not relevant.*

A comparison between the integrated response under the standard reference spectrum and the response measure under the simulator

☒ Yes — The integrated Jsc from EQE were agree well with the Jsc determined from the J-V measurements
☐ No — *Explain why this information is not reported/not relevant.*

For tandem solar cells, the bias illumination and bias voltage used for each subcell

- [x] Yes
- [ ] No

Stated in Methods section.

Explain why this information is not reported/not relevant.

5. Calibration

Light source and reference cell or sensor used for the characterization

- [x] Yes
- [ ] No

Stated in Methods section.

Explain why this information is not reported/not relevant.

Confirmation that the reference cell was calibrated and certified

- [x] Yes
- [ ] No

The output intensity of the light source was calibrated using the short-circuit current of a standard monocrystalline silicon (Si) solar cell from Fraunhofer ISE.

Explain why this information is not reported/not relevant.

Calculation of spectral mismatch between the reference cell and the devices under test

- [x] Yes
- [ ] No

The spectra mismatch between the reference cell and the devices under test were used to adjust the intensity of solar simulator lamp.

Explain why this information is not reported/not relevant.

6. Mask/aperture

Size of the mask/aperture used during testing

- [x] Yes
- [ ] No

0.15 cm2

Explain why this information is not reported/not relevant.

Variation of the measured short-circuit current density with the mask/aperture area

- [ ] Yes
- [x] No

Report the difference in the short-circuit current density values measured with the mask and aperture area.

Our results are always reported with aperture.

7. Performance certification

Identity of the independent certification laboratory that confirmed the photovoltaic performance

- [x] Yes
- [ ] No

Quality Supervision & Testing Center of Chemical & Physical Power Sources of the Information Industry (QSTC)

Explain why this information is not reported/not relevant.

A copy of any certificate(s)

- [x] Yes
- [ ] No

Provided in the Supplementary Note 1.

Explain why this information is not reported/not relevant.

8. Statistics

Number of solar cells tested

- [x] Yes
- [ ] No

Stated in the manuscript.

Explain why this information is not reported/not relevant.

Statistical analysis of the device performance

- [x] Yes
- [ ] No

Fig. 1b-e, Fig. 3a-d, Supplementary Figs. S10, S11, S15, S25, S26, and S28.

Explain why this information is not reported/not relevant.

9. Long-term stability analysis

Type of analysis, bias conditions and environmental conditions

- [x] Yes
- [ ] No

Fig. 4b, Fig. 4c, and Fig. 4d.

Explain why this information is not reported/not relevant.
